# Tree Productivity Enhanced with Conversion from Forest to Urban Land Covers

**DOI:** 10.1371/journal.pone.0136237

**Published:** 2015-08-24

**Authors:** Brittain M. Briber, Lucy R. Hutyra, Andrew B. Reinmann, Steve M. Raciti, Victoria K. Dearborn, Christopher E. Holden, Allison L. Dunn

**Affiliations:** 1 Department of Earth and Environment, Boston University, Boston, Massachusetts, United States of America; 2 Department of Biology, Hofstra University, Hempstead, New York, United States of America; 3 Department of Earth, Environment, and Physics, Worcester State University, Worcester, Massachusetts, United States of America; Peking University, CHINA

## Abstract

Urban areas are expanding, changing the structure and productivity of landscapes. While some urban areas have been shown to hold substantial biomass, the productivity of these systems is largely unknown. We assessed how conversion from forest to urban land uses affected both biomass structure and productivity across eastern Massachusetts. We found that urban land uses held less than half the biomass of adjacent forest expanses with a plot level mean biomass density of 33.5 ± 8.0 Mg C ha^-1^. As the intensity of urban development increased, the canopy cover, stem density, and biomass decreased. Analysis of *Quercus rubra* tree cores showed that tree-level basal area increment nearly doubled following development, increasing from 17.1 ± 3.0 to 35.8 ± 4.7 cm^2^ yr^-1^. Scaling the observed stem densities and growth rates within developed areas suggests an aboveground biomass growth rate of 1.8 ± 0.4 Mg C ha^-1^ yr^-1^, a growth rate comparable to nearby, intact forests. The contrasting high growth rates and lower biomass pools within urban areas suggest a highly dynamic ecosystem with rapid turnover. As global urban extent continues to grow, cities consider climate mitigation options, and as the verification of net greenhouse gas emissions emerges as critical for policy, quantifying the role of urban vegetation in regional-to-global carbon budgets will become ever more important.

## Introduction

Terrestrial ecosystems are an important and dynamic component of the global carbon cycle. In recent decades, terrestrial ecosystems have sequestered approximately 25% of the carbon emitted to the atmosphere by human activities [1]. Patterns of human development, including deforestation and urbanization, have changed the spatial distribution, structure, and extent of terrestrial ecosystems. Understanding the consequences of urban land use and land cover changes on terrestrial productivity and carbon stocks is critical for modeling the local, regional and global carbon cycle [2,3].

Population growth, migration into cities, and sprawling forms of land development are increasing urban populations and the spatial extent of cities. Between 1950 and 2010, the global population grew from 2.6 to 6.9 billion people, with the percentage of people living in cities increasing from 29% to 52% [4]. Urban areas comprise nearly 3% of global land area [5], an extent comparable to that of temperate forests [6], and the urban extent is projected to double by 2050 [4]. In the U.S., approximately 80% of the population now lives in urban areas [7] and urban land cover expanded by 50–380% between 1974 and 2002, depending on how urban land cover is defined [8–10].

Urban land cover is inherently heterogeneous, with roads, buildings, and vegetation co-occurring within small patches of land. Urban areas can include significant quantities of vegetation [11], and often have a complex land use history [12]. These areas represent a patchwork of vegetated and developed land covers across which ecosystem function, productivity, and structure vary significantly [13,14].

The global environmental impacts of cities are growing [15–17]. Cities are estimated to consume 67% of global energy and emit 71% of energy-related carbon emissions [18]. Further, urban development results in additional carbon emissions associated with the clearing and paving of land [17,19,20]. Studies also suggest that urban areas have unique meteorological and atmospheric properties [21], distinct flora and faunal diversity [22], and higher levels of air and water pollution due to increased human activity and waste production [16]. These ecological impacts of urbanization extend far beyond individual city limits through environmental teleconnections and demand for agricultural and energy related goods and services [23,24].

Vegetation in urban areas experiences modified growing conditions [14,25]. Urban areas typically exhibit 3–12°C higher mean annual temperatures than adjacent rural locations [26–28] and this “urban heat island” effect has been shown to increase growing season length by 15–31 days in New England cities [29,30]. Fertilizer applications and enhanced urban atmospheric N deposition have been associated with increased soil N concentrations [31]. Atmospheric N inputs have been positively correlated with on-road CO_2_ emissions [32] and proximity to major roadways [33], highlighting the influence of human activity on plant nutrient inputs. Furthermore, urban areas often exhibit atmospheric CO_2_ concentrations that are 10–50 ppm above ambient, which can stimulate plant productivity [29,34,35]. Elevated concentrations of urban atmospheric aerosols increase diffuse beam radiation, thereby potentially enhancing photosynthetic efficiency of vegetation [36,37]. While increases in N inputs, atmospheric CO_2_, and light availability coupled with a longer growing season may enhance plant productivity in urban areas, carbon deficient and compacted soils [38–40], foliar ozone damage [41], and reduced water availability due to runoff and the presence of hydrophobic soils [13] may limit plant productivity. Our understanding of the net effect of urbanization on plant growth remains uncertain. The few existing *in situ* studies focusing on urban vegetation productivity found higher growth rates for urban trees than for trees in adjacent rural areas [42,43]. These studies focused on seedlings and saplings and did not consider land use history in their sampling.

In this study we assessed the effect of land cover conversion from forest to a variety of urban land covers on vegetation dynamics. Specifically, we used a combination of remote sensing, field biometric measurements, dendrochronology, and tax assessor records to quantify how vegetation structure and growth rates changed as a function of urban development type and development intensity. We hypothesized that urbanization would simultaneously decrease terrestrial carbon pools and increase the relative ecosystem productivity of the remaining vegetation.

## Materials and Methods

### Study Area

**Fig 1:**
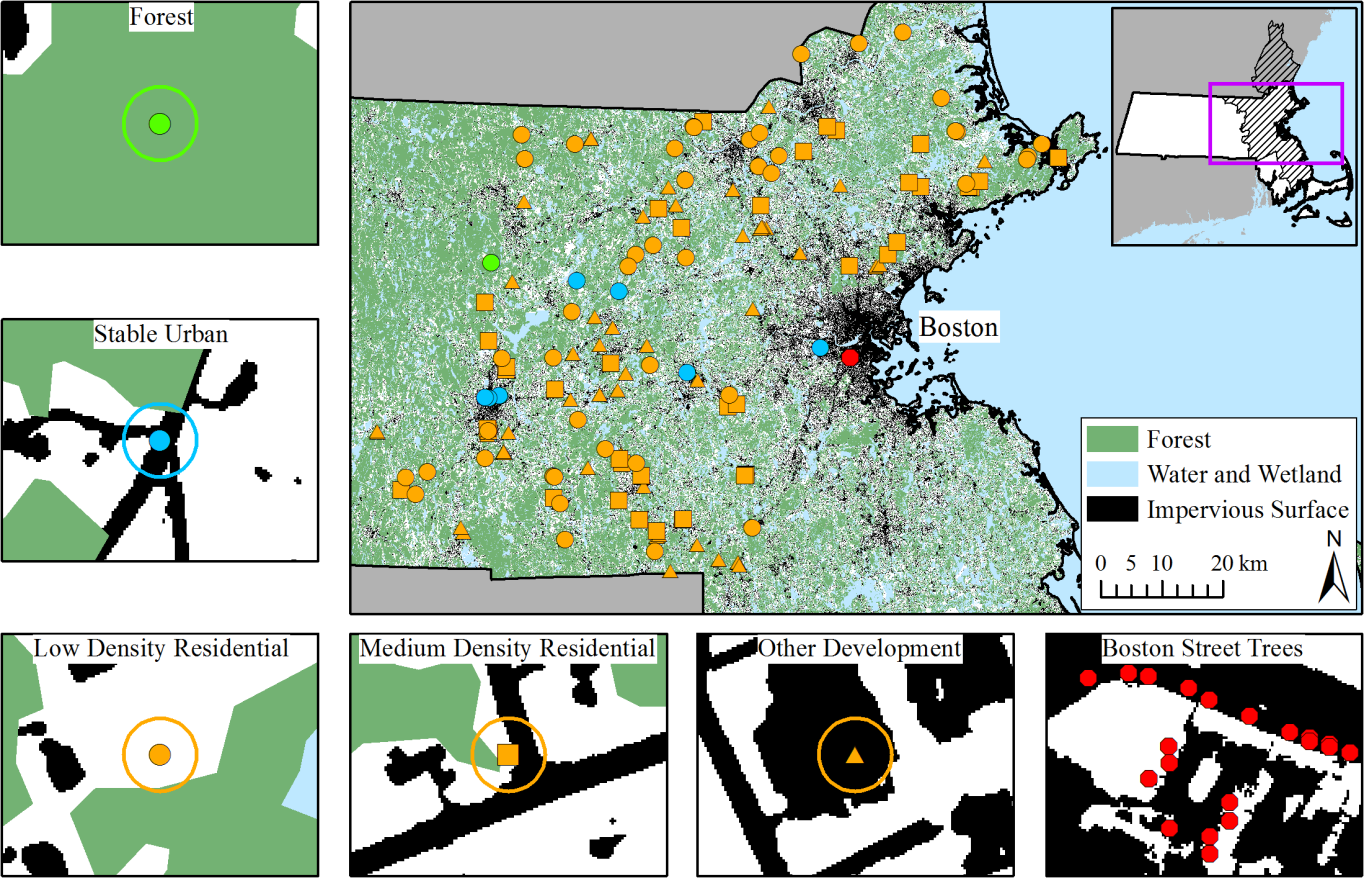
The study area. The maps show the distribution of forested land use/cover and impervious surfaces across eastern Massachusetts. The hatched area on the inset denotes the extent of the Boston MSA. Each point on the map reflects field plot locations and the area for street tree sampling. All of the orange points were converted from forest to urban land uses between 1971 and 2012; the shapes of the points denote the intensity of urban development, which include low density residential (LDR; circle), medium density residential (MDR; square), and other development (OTH; dominated by commercial, industrial, high density residential, and parking lot areas; triangle). The green, blue, and red points indicate plot locations for stable forests, stable urban land covers, and City of Boston street trees, respectively. The panels surrounding the central map illustrate the land cover characteristics of the different plot types, with white areas representing all other land covers (e.g. lawns, isolated trees, etc.). Example field plots are shown to scale (30 m diameter) and point locations for street trees correspond to sample trees.

This study was conducted across a range of land covers in eastern Massachusetts (Fig 1). Boston is the largest urban area in New England, both in terms of population and land extent. The population within the Boston Metropolitan Statistical Area (MSA) is approximately 4.6 million, making it the 10^th^ largest MSA in the United States. Since 1950, the Boston MSA population has increased by an average of nearly 1% yr^-1^ [44]. The region has a humid continental climate (Köppen classification Dfa), which is characterized by cold, snowy winters and hot, humid summers: Boston’s mean January and July temperatures are -1.5° and 23.3°C, respectively. There is a 0.25–1.5°C mean annual temperature gradient between our coastal and inland field sites. Mean annual precipitation is 1056 mm and is evenly distributed throughout the year [45]. Vegetation in eastern Massachusetts is dominated by temperate mixed-hardwood forests that include red oak (*Quercus rubra*), white pine (*Pinus strobus*), red maple (*Acer rubrum*), and paper birch (*Betula papyrifera*) [46]. Soils in eastern Massachusetts are glacial in origin and are dominated by sand and silt loams and, to a lesser extent, rocky outcroppings [47].

Land use and land cover in eastern Massachusetts vary considerably and reflect evolving land use practices and development patterns over the last several hundred years. Most of the forested land in eastern Massachusetts was cleared for agriculture or human settlement in the 19^th^ century. Agricultural abandonment in the early 20^th^ century resulted in widespread reforestation across the region [48]. While eastern Massachusetts has a large and dense population, it also includes patches of extensive vegetation cover, with 41.7% of the Boston MSA forested [49].

To quantify changes in vegetation structure and productivity that occur after urban development, we established 135 field plots (706.9 m^2^ each) across eastern Massachusetts. All plots were located on land with a coarse-loamy, mixed, mesic typic dystrochrepts soil, the most common soil family in eastern Massachusetts [47]. All of these plots were converted from forest to an urban land cover between 1971 and 2012. We also revisited existing plots from Raciti et al. [49] that were on the same soil family and had red oak present. Additionally, and in collaboration with the Morton Arboretum, we analyzed street tree growth rates in the City of Boston (Scharenbroch, unpublished data). For the purposes of data comparison and extrapolation, our final study area included five eastern and central Massachusetts counties: Essex, Suffolk, Middlesex, Norfolk and Worcester.

### Plot Selection and Stratification

Definitions of “forest” and “urban” areas vary geographically and with the context of analysis (e.g. Raciti et al. [49]). For the purposes of this analysis, we used two different remote sensing-based land use and land cover change datasets to identify candidate locations for field sampling and categorize land cover. The MacConnell dataset was based on manual photo interpretation and included land use maps for 1971, 1985, and 1999 [50]. In addition, we used the Continuous Change Detection and Classification (CCDC) [51] algorithm applied to a timeseries of Landsat observations in order to identify changes between 1984 and 2012. Combining the two datasets provided a forty-year time window for land cover change analysis.

The MacConnell land use maps were created by classifying 21 distinct land covers/uses using 1:25,000 aerial photographs across Massachusetts. The resulting data layers were developed explicitly for land cover change analysis and contain nested polygons at a 0.4 ha minimum mapping unit, with each polygon containing classification values for all three time periods. Polygons containing change after 1971 were subset and relabeled in a nested, hierarchical fashion to allow direct comparison across time periods. Using the CCDC algorithm, we analyzed the spectral reflectance from all available Landsat observations between 1984 and 2012 at each 30m pixel to identify the timing of spectral disturbances. We categorized change in land cover by classifying the land cover of stable time periods before and after a disturbance and comparing pixel-level reflectances to their stable model statistics [51]. These land cover change products were combined to stratify field plots based on the intensity of urban development and time since development.

The MacConnell and CCDC products had different mapping units (0.4 ha and 0.09 ha, respectively) and different output data given their focus on land use and land cover, respectively. To facilitate comparison, the CCDC maps were filtered to include only land cover change pixels that were neighbored by at least two additional change pixels, changing from forest to any type of developed land cover. Filtering by such pixel groups helped to reduce high frequency errors and spectral changes that did not represent actual land cover change while increasing the effective minimum mapping unit to a level comparable with the MacConnell maps. In addition, we spatially dilated the area of land cover change when there were three or more change pixels adjacent to one another. Dilating areas of land cover change identified by CCDC reduced change pixel omission along the edges of these patches and created continuous polygons that more closely reflect the land use change polygons mapped by MacConnell. For example, while forest edges adjacent to new suburban developments might not have changed their spectral reflectance—and thus would not have been picked up by CCDC—the land use may change from a forest to a developed, residential use.

Using land use and land cover change locations identified by the MacConnell and CCDC maps, we stratified field plots into three urban intensity categories; low density residential (LDR), medium density residential (MDR), and other development (OTH). These classifications, which were generated before fieldwork began, were also used to analyze and parse processed data. These classes were defined by combining a collapsed version of a Landsat based Massachusetts training dataset [51] with a 2005 1m impervious Massachusetts GIS data layer [52]. Areas around and including the LDR plots exhibited 0–50% impervious surface area (ISA) which contained single family residences; areas around and including the MDR plots exhibited 0–80% ISA and typically contained multi-story and condominium residences; areas around and including the OTH plots exhibited greater than 80% ISA and were dominated by parking lots and commercial and industrial buildings. There was overlap in the ISA range for the LDR and MDR classes, but the land use composition of those classes differed in housing density. The urban categorization was confirmed with field observation. All field plots converted from forest to urban development and were further stratified into three time windows of conversion: 1971–1985, 1985–1999, and 1999–2012. This sample design resulted in nine different land use and time since land cover change categories.

With a goal of sampling at least 15 plots in each of these nine change categories, we generated a stratified random sample of 1,500 candidate plot locations from both public and private lands sharing the same soil type. Following the methods outlined in Raciti et al. [49], we used digital parcel data to derive ownership information and mailed letters requesting permission to sample. We visited only those sites for which permission to conduct the study on that site was granted, including sites on public and private property. Access limitations prevented us from using a truly random sample. In order to prevent a potential bias towards plots where access was easier (e.g., commercial parking with no vegetation), we sampled public and private properties in the same proportion as found among the first 15 randomly chosen plots in each category. The final number of plots in this “land cover change” group included a minimum of 14 plots in each of the nine categories for a total of 135 plots (Fig 1). For most of the analysis, the plots were aggregated based on the land use conversion category: forest-to-LDR, forest-to-MDR, and forest-to-OTH. The minimum number of plots in each of these three categories was 43.

### Field Sampling

Field sampling took place from June through August 2013. Each sample plot was circular with a 15 m radius (706.9 m^2^). Plot centers were located using satellite basemaps in ArcMap 10.0 (ESRI, Redlands, CA) and handheld GPS units with accuracies of ~3 m. A TruPulse 200 Professional Range Finder and Hypsometer (Laser Technology, Centennial, CO, U.S.) and field measuring tapes were used to determine the plot boundaries. The tree canopy cover was visually estimated from the ground at each plot and ground cover fraction was visually estimated for lawn, garden, forest, degraded forest (defined as small, unmanaged tracts that were often surrounded by development), weedy, bare ground, and paved surfaces.

All trees with a diameter at breast height (DBH; measured at 1.37 m aboveground) ≥ 5 cm were measured and identified to species, whenever possible. Coarse woody debris (CWD) with a minimum diameter of 10cm and 1m length were measured within the plot area using either a Haglöfs (Langsele, Sweden) 40 cm analogue caliper or a DBH tape measure. See Raciti et al. [49] for a complete methodological description of the aboveground live and dead biomass estimations.

### Tree Cores

We extracted one tree core in each plot at breast height from a red oak ≥ 20 cm in DBH with a 200 mm long Haglöfs 5 mm increment borer. If no red oaks were present within a sample plot, cores were extracted from a similarly sized red oak within ~50m of the plot center. Only one core was extracted from each plot due to property owner concerns about tree damage. In an attempt to control for differences in light conditions across plots, we preferentially extracted cores from canopy trees that were located along forest edges. These trees frequently had crowns that were directly abutted on one side by other trees’ crowns. Cores were mounted and glued onto grooved wood blocks in the field.

A total of 75 tree cores were extracted. After drying, each core was sanded on a belt sander to a broad, flat surface using coarse, 220 grit sand paper and then sanded by hand with fine, 400 grit paper to create a smooth surface. Cores were scanned using a high-resolution color scanner (Epson Perfection V700 Photo) and ring increments were measured using WinDENDRO 2012 (Regent Instruments, Inc., Sainte-Foy, Quebec, Canada) image analysis software. All increment measurements were independently verified for their ring count and position by at least two observers. When chronologies were long enough, initial cross-dating was performed by identifying statistically significant reductions in growth during the gypsy moth outbreak of 1980 [53]. All measurements, regardless of chronology length, were cross-dated using the program COFECHA [54,55].

DBH (cm) for each year in a core’s chronosequence was calculated as

$$DBH_{i}=DBH_{2013}–2*RD_{i}$$ (1)

where DBH_2013_ is the DBH measured in 2013 DBH and RD is the radial distance from the year i to the bark. Biomass was calculated using a forest-based red oak allometric equation [56] with biomass in kg and DBH in cm:

$$Biomass=0.113\;*\;\left(DBH^{2.4572}\right)$$ (2)

Basal area was estimated as follows with basal area in cm^2^ and DBH in cm:

$$Basal\;area=\pi *{\left(DBH/2\right)}^{2}$$ (3)

Basal Area Increment (BAI) and biomass increment (BI) were calculated for each year (Y) as:

$$BAI_{Y}=basal\;area_{Y}-basal\;area_{Y-1}$$ (4)

$$BI_{Y}=biomass_{Y}-biomass_{Y-1}$$ (5)

where the units of BAI and BI are cm^2^ yr^-1^ and Kg yr^-1^, respectively.

Land cover change dates for each core were determined using a combination of Massachusetts parcel data from tax assessor records [57], remote sensing CCDC results [51], and aerial imagery from Google Earth [58]. In cases where the tax assessor record was incomplete, we used alternative commercial real estate databases to supplement the parcel records (www.thewarrengroup.com, www.zillow.com). In addition, time-lapse imagery in Google Earth was used to confirm and identify land cover changes for each plot between 1993 and 2012.

We required a minimum of five years of increment data before and after land cover change for each core to obtain robust growth estimates. We assessed the suitability of requiring 10 years of rings before and after development, but found that 1) this reduced the number of available cores from 59 to 46; 2) the 10 year window yielded aggregated before and after BAI means within 2% and 18%, respectively, of those using the 5 year window; and 3) the 5-year window acceptably captured typical inter-annual variability (S1 Fig). Of the 75 cores extracted in 2013 for analysis in the “land cover change” category, four cores were excluded from analysis as they did not have a minimum of five years of growth increment data both before and after the established land cover change date and nine additional cores were removed due to severe rot and/or unreadable rings. Five cores were reclassified from the land cover change category to the “stable urban” category (see below) as these plots likely had no vegetation pre-dating land cover change or we could not locate any physical records that the plot had been modified during the last 50 years. Two cores from the Raciti et al. [49] sampling effort were found to have converted from forest-to-LDR since 1971 and were reclassified accordingly. In total, we had 59 useable cores for our land cover change analysis, 57 of which came from our 2013 field campaign and two of which came from our 2010 field campaign.

### Background, Stable Urban, and Street Tree Increment Data

In order to develop background regional tree growth rates for forest trees we used Forest Inventory and Analysis data [59] from 99 red oaks within our study area. The spatial distribution of these FIA data were not as even as the spatial distribution of our land cover change cores: 82 of the FIA data originated from trees in Worcester County, while the remaining 11 came from locations north and northwest of Boston. Annual increment data was not available from the FIA; we estimated an average increment by dividing the most recent DBH by the tree age.

Tree growth rates for street trees within the City of Boston were estimated using 17 red oak cores collected by the Morton Arboretum in 2012 (Scharenbroch, personal communication). The Boston street trees were all open grown, and most were in high exposed light environments. The street tree cores were prepared (mounted, sanded, etc.) and scanned by the Morton Arboretum; we processed the images with WinDENDRO using the methods described above.

We also obtained tree growth rates for our “stable urban” category by revisiting 13 plots from the work described in Raciti et al. [49]. Eleven of these plots did not undergo land cover or use change from 1971 to 2012 and were classified as stable urban. The two plots that underwent recent forest to urban land cover change were analyzed with our 2013, land cover change data. Including the four reclassified cores from our 2013 field campaign, we analyzed a total of 15 stable urban cores. One of the 15 plots was also identified as being completely forested (see Fig 1).

All 2010 and 2013 plots, FIA, and Boston street tree data originated from within 55 miles of Boston. What distinguished our urban, land cover change plots from our background, forest plots was that our urban plots were in locations that had experienced anthropogenic land cover change. A list of all plots from the land cover change, stable urban, Boston street tree, and FIA categories with location information is provided in the supplemental materials (S1 Table).

### Statistical Analysis

Statistical analysis was conducted using R Statistical Software 3.1.3 [60]. Unless specifically noted otherwise, all reported errors in the text and figures reflect 95% confidence intervals. Confidence intervals were calculated by bootstrap analyses [61] due to heteroscedasticity within the data distributions. Bootstrap samples were drawn 1000 times with replacement to estimate 95% confidence intervals around mean. Error estimates do not include allometric or spatial scaling errors. An alpha-value of 0.05 was used to denote significance.

## Results

### Plot Biomass, Ecosystem Structure, and Land Cover in Urban Plots

**Fig 2:**
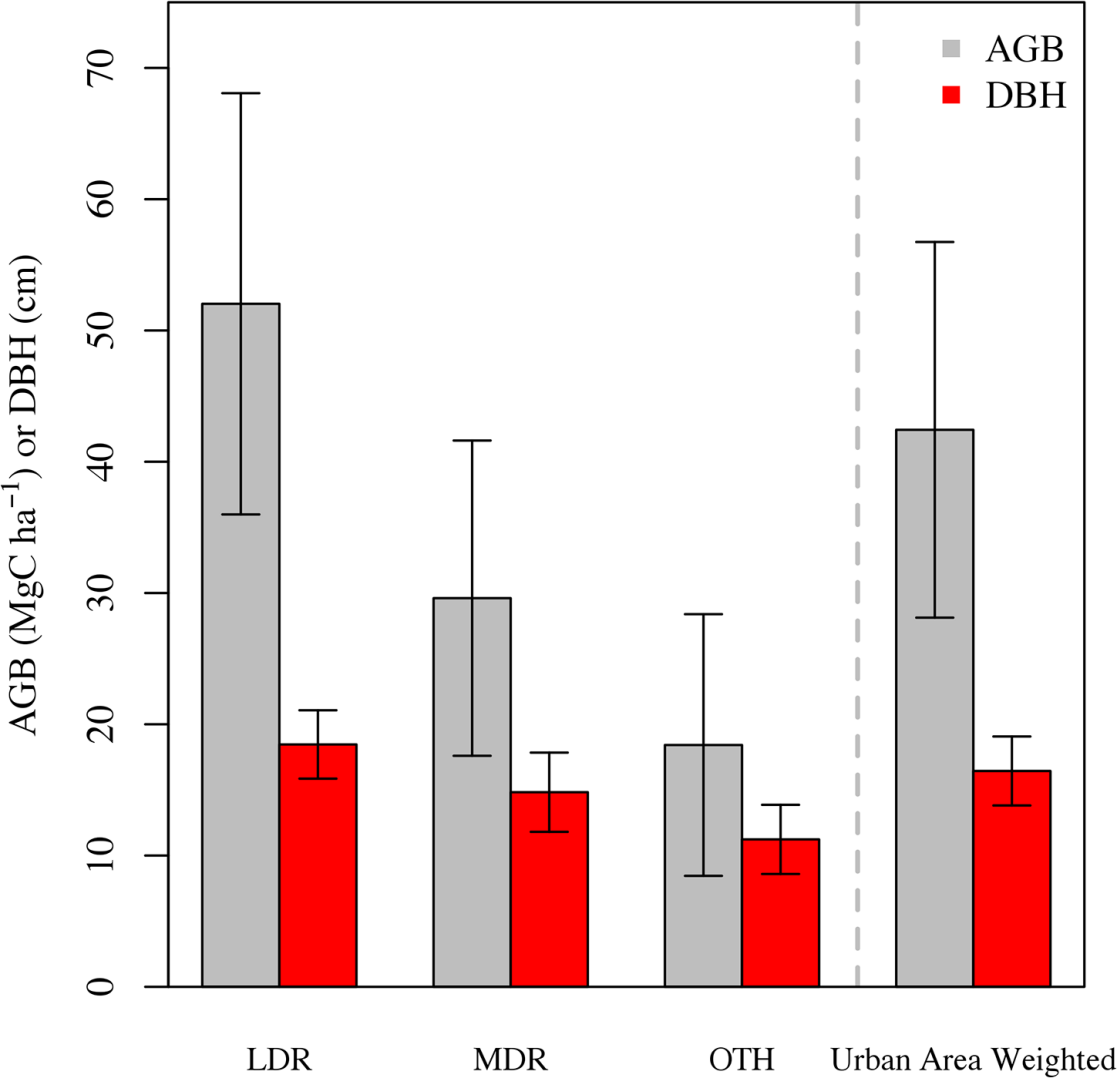
Ecosystem structure. Aboveground biomass (AGB) and diameter at breast height (DBH) for all low density residential (LDR), medium density residential (MDR), and other (OTH) 2013 land cover change field plots. The area weighted urban estimate was based on the areal extents from Table 1. Error bars are 95% confidence intervals.

Mean plot-level DBH, total live aboveground biomass, stem density, and canopy cover all declined as development intensity increased such that LDR > MDR > OTH (Table 1 and Fig 2). Across all forest-to-urban land cover conversion pathways, we found a mean, area-weighted aboveground biomass of 42.7 ± 14.7 Mg C ha^-1^, approximately 54% of the mean eastern MA forest biomass (78.6 Mg C ha^-1^) reported by the FIA [59]. While the changes in ecosystem structural characteristics were broadly consistent across all land cover change categories, differences between the forest-to-LDR and forest-to-MDR categories were more pronounced than differences between the forest-to-MDR and forest-to-OTH categories (Table 1). Moreover, as development intensity increased from LDR to OTH, aboveground biomass decreased by about 65% from 52.3 ± 16.0 to 18.4 ± 9.5 Mg C ha^-1^, respectively. Biomass differences were more pronounced than accompanying differences in canopy cover and stem density across the same change in development intensity.

**Table 1:** Ecosystem characteristics and land conversion rates. Ecosystem structure characteristics of all plots, parsed by forest conversion pathway. Land area within the eastern Massachusetts study area includes all five counties in which we had study plots and is approximately 8,800 km^2^. Stem density and canopy cover estimates across all development types are area weighted based on the current areal extent of each development category. Land conversion rates were based on the difference in areal extent within each category between 1971 and 1999. Current values were generated from 2013 field and FIA data.

	Land Conversion Rate (km^2^ yr^-1^)	Current Areal Extent (km^2^)	Percent of Study Area	Number of Plots	Current Stem Density (stems ha^-1^)	Current Canopy Cover (%)
**Forest-to-LDR (0–50% ISA)**	20.3	2028	23.0	46	359 ± 109	45.2 ± 9.7
**Forest-to-MDR (0–80% ISA)**	1.3	110	1.3	43	241 ± 96	30.0 ± 9.8
**Forest-to-OTH (>80% ISA)**	0.9	75	8.5	46	232 ± 94	25.2 ± 10.4
**All Development Types**	22.5	2890	32.8	135	322 ± 99	39.4 ± 9.9

We found that trees ≥ 20cm in DBH (minimum size for coring) had a stem density of 106.1 ± 18.3 stems ha^-1^, represented an area-weighted mean biomass of 39.1 ± 7.7 Mg C ha^-1^, and had a mean post-development growth rate of 16.8 ± 0.6 kg C tree^-1^ yr^-1^. Excluding stem recruitment, planting, and mortality and assuming all trees exhibit the same growth response to development as those cored for this study, our data suggest an aboveground biomass growth rate of 1.8 ± 0.4 Mg C ha^-1^ yr^-1^, representing a 4.6% yr^-1^ increase in biomass (ranging from 3 to 7% yr^-1^).

Visual estimates of ground cover were highly variable across all urban land cover types. As development intensity increased from LDR to OTH development types, plot-level lawn extent showed no statistically significant change. However, there was a significant increase in plot-level ISA from 20.2 ± 6.8% to 53.0 ± 10.2% [52] as development intensity increased from LDR to OTH development types. We found no significant relationship between the amount of ISA on the plot and stem density, live biomass, or tree-level BAI.

Land cover conversion rates between 1971 and 1999 indicate reductions in forest cover that are concomitant with increases in the coverage of urban development, especially in the form of forest-to-LDR land cover change (see Table 1). Between 1971 and 1999 forest cover within the study area decreased 13% from 5,023 to 4,451 km^2^ [50]. Given that most of this forested land was converted to some kind of urban development, we estimated that the resulting forest area lost was roughly 0.23% yr^-1^ [50]. However, independent, county-level FIA estimates suggest that forest area loss may have been higher at 0.35% yr^-1^ for 1985–1998, decreasing to 0.19% yr^-1^ between 2005 and 2012 [62]. Based on the 2011 National Land Cover Database, forested and developed land covers (LDR, MDR, OTH) comprised approximately 54% and 33%, respectively, of total land cover within our study area [63].

### Tree Growth Rates

We analyzed a total of 91 tree cores for annual growth increment. Cores from plots that underwent conversion from forest-to-urban land covers since 1971 (n = 59 cores) had a mean DBH at the time of sampling of 45.6 ± 3.2 cm, with a mean BAI of 25.2 ± 3.7 cm^2^ yr^-1^. Cores from open-grown Boston street trees (n = 17 cores) had a mean DBH of 39.6 ± 6.5 cm at the time of sampling, with a mean BAI of 36.9 ± 9.5 cm^2^ yr^-1^. Cores from the stable urban plots that did not undergo any land cover change since 1971 (n = 15 cores) had a mean DBH of 45.9 ± 9.2 cm at the time of sampling, with a mean BAI of 30.0 ± 7.1 cm^2^ yr^-1^. Data for the background growth category were based on 99 trees from FIA plots with a mean DBH of 31.4 ± 1.2 cm at the time of sampling and a mean BAI of 13.4 ± 1.1 cm^2^ yr^-1^.

**Fig 3:**
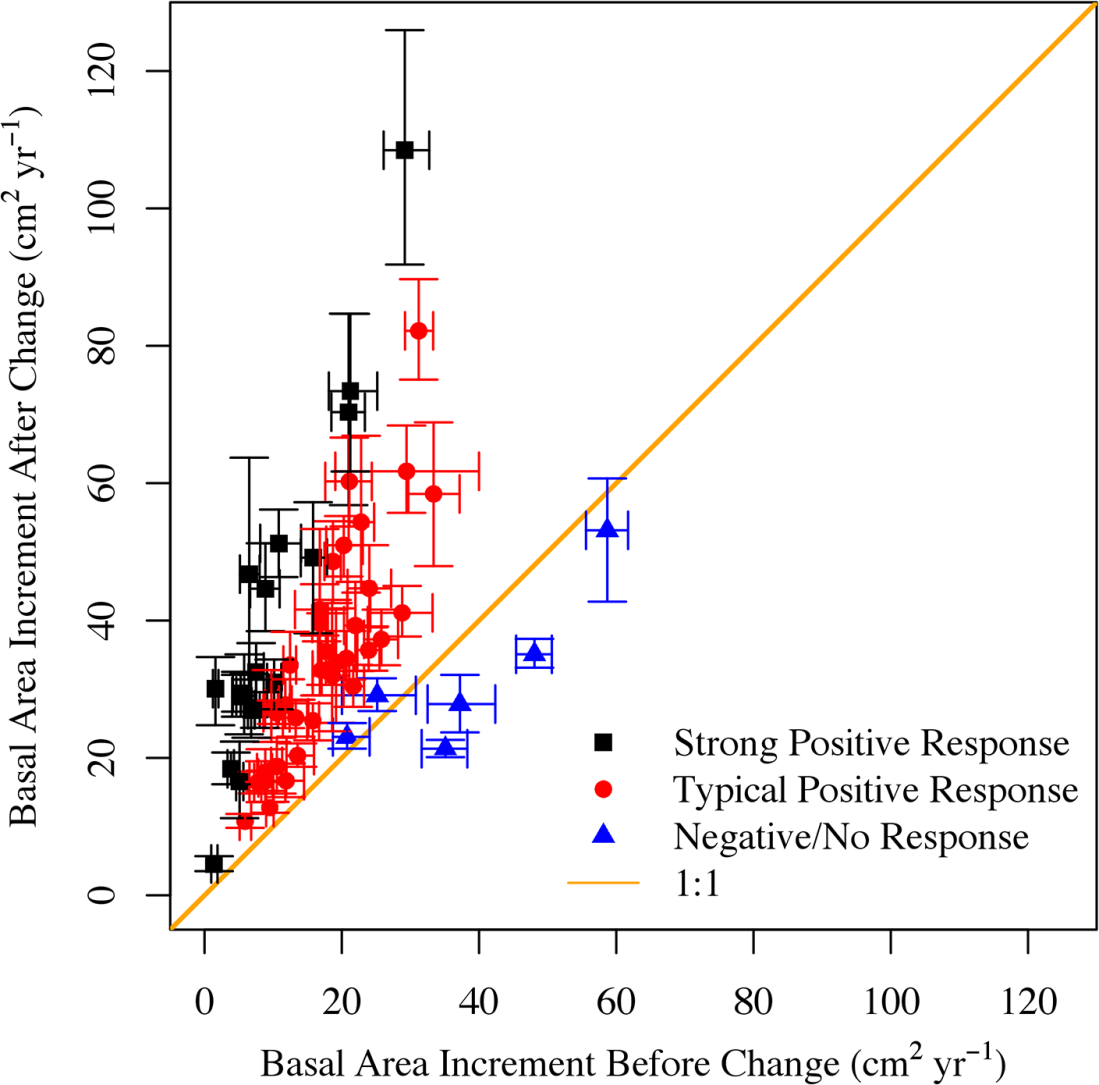
Basal area increment before and after land cover change. Mean basal area increment before and after conversion from forest to urban land cover for each tree. The 95% confidence intervals reflect both inherent variability in growth rates and changes in the number of rings before and after land conversion. Individual cores are color coded to denote the difference in before vs. after basal area increment and are categorized as a “Negative/No Response”, “Typical Positive”, and “Strong Positive.” Trees which exhibited a “strong” response were those that had a ≥ 200% increase in growth rates following land use change.

Of the 59 cores analyzed from sites that underwent land cover change, there were 28, 18, and 13 cores in the LDR, MDR, and OTH subcategories, respectively. Differences in increment growth rates between development trajectories (i.e., urban subcategories) were not statistically significant and were aggregated for analysis. Following conversion of forest to urban development, the mean overall BAI increased from 17.1 ± 3.0 cm^2^ yr^-1^ to 35.8 ± 4.7 cm^2^ yr^-1^ (Fig 3). Fifty-three of the 59 tree cores showed a significant increase in BAI, while 18 cores showed an increase of over 200% after conversion (9.6 ± 3.9 to 39.7 ± 11.7 cm^2^ yr^-1^). Generally, the cores with a lower initial BAI showed a larger growth response following development (Fig 3). Three cores showed no significant change and three cores showed a decrease in growth post-development. Overall mean annual biomass increment increased by 138%, from 6.5 ± 1.6 to 16.9 ± 3.0 kg C tree^-1^ yr^-1^ after the land conversion date.

**Fig 4:**
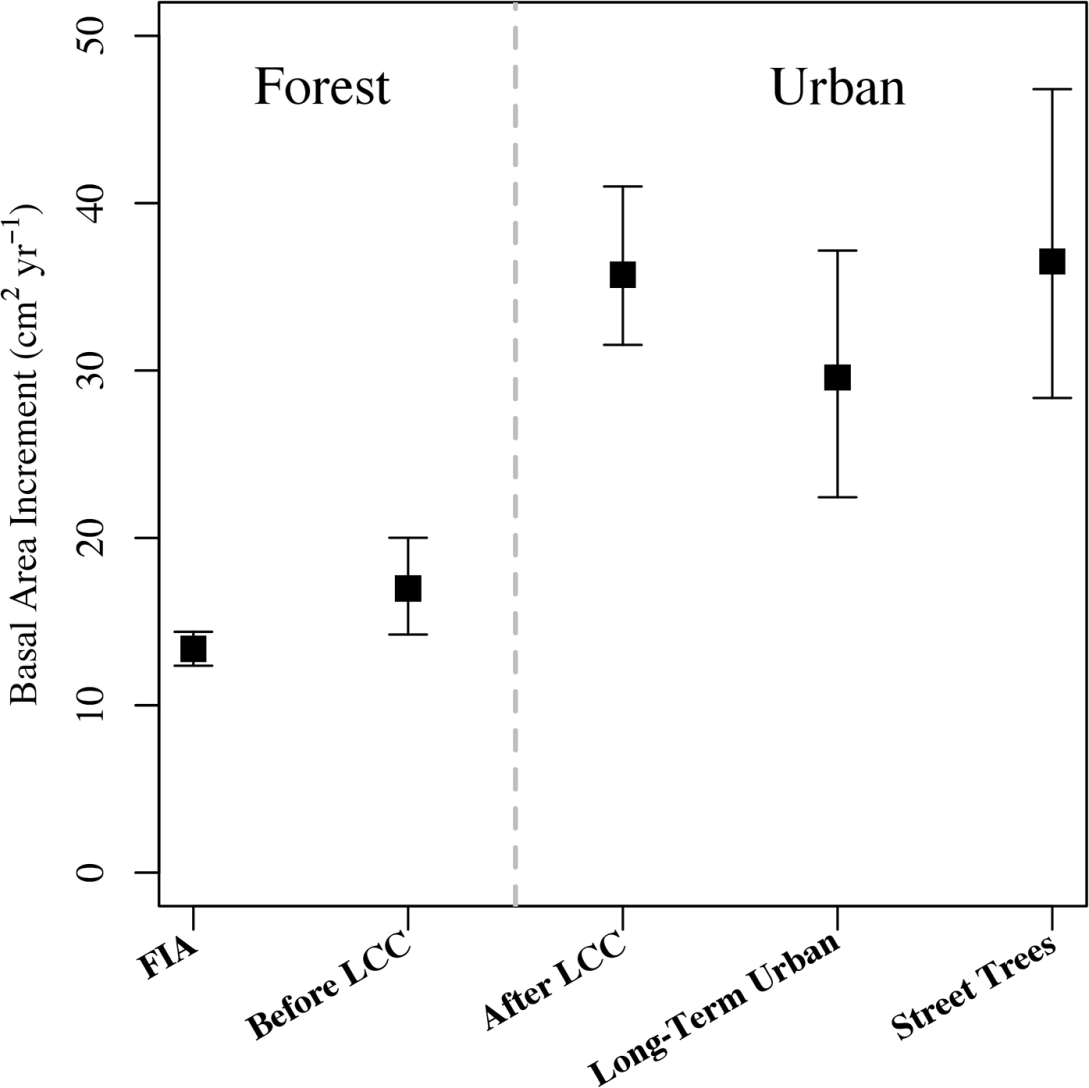
Basal area increment for forest and urban land covers. Mean Basal Area Increment (BAI) within forested areas was based on the FIA regional plots and growth rates before land cover change. Urban BAI includes growth rates after development, trees from stable urban plots, and open-grown Boston street trees.

Across all cores, the average BAI indicated an increase in productivity after land cover change (Fig 4). The mean growth rates for the period after land conversion, street trees, and stable urban plots were similar at 35.8 ± 4.7, 36.9 ± 9.5, 30.0 ± 7.1 cm^2^ yr ^-1^, respectively. Annual growth rates from these urban sites were approximately double the growth rates of the forest-grown red oaks sampled from the FIA plots and the pre-development BAI. Estimated growth rates in the FIA trees (13.4 ± 1.1 cm^2^ tree^-1^ yr^-1^) were similar to the pre-conversion growth rates at our sites undergoing land cover change (17.1 ± 3.0 cm^2^ tree^-1^ yr^-1^).

**Fig 5:**
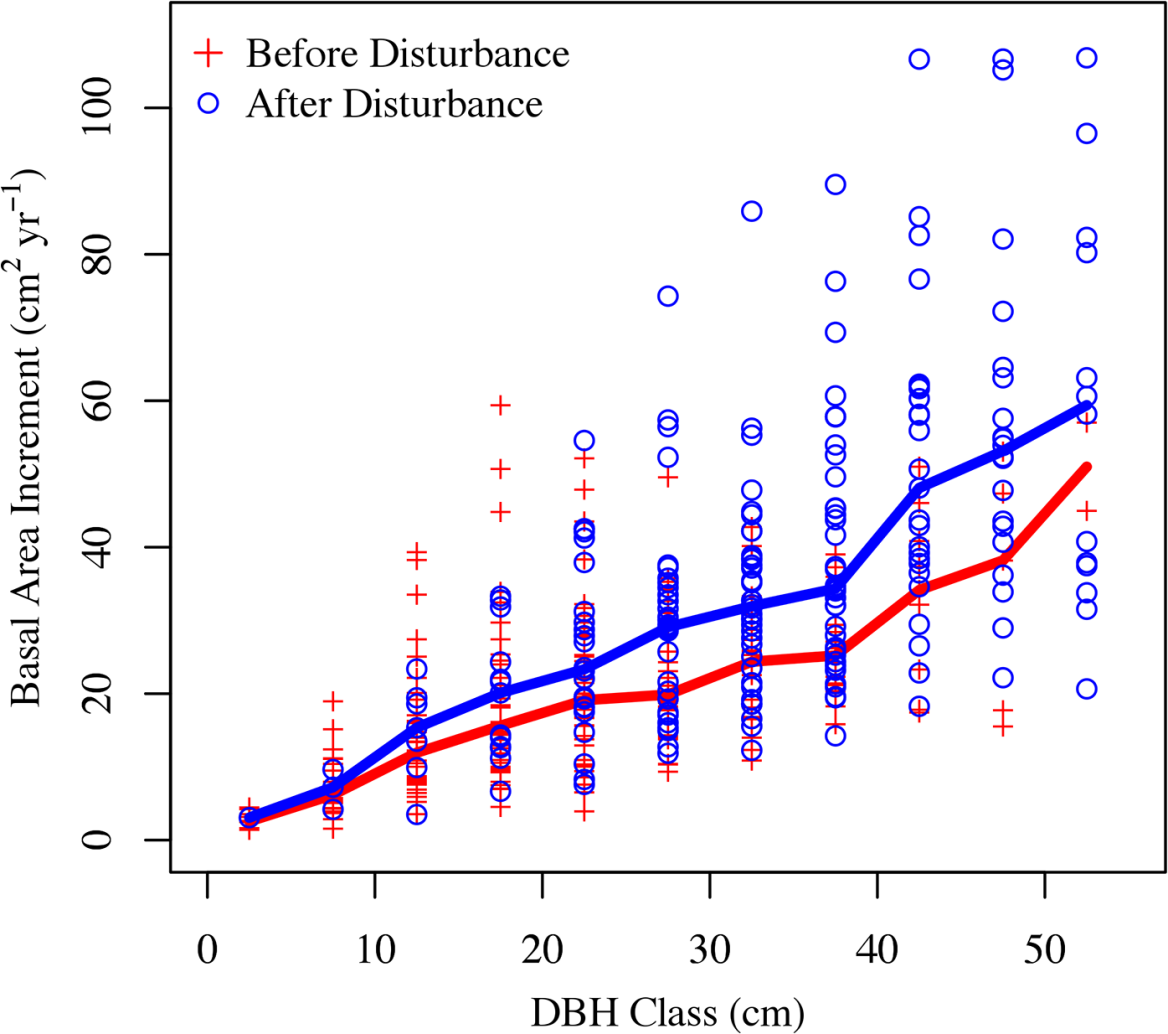
Basal area increment as a function of DBH class. Basal area increment as a function of diameter at breast height (DBH) class before and after conversion to urban land covers. DBH classes represent binned DBH values spaced at 5cm intervals. The solid lines represent the medians for each category.

The mean DBH at the time of conversion was 28.5 ± 3.6 cm, while the mean DBH at the time of sampling (i.e., 2013) was 45.6 ± 3.2 cm. To test for tree size-related biases in the observed before-after land cover change BAI, we also calculated BAI as a function of size and found that, on average, trees within a given DBH class grew faster after the conversion than before the conversion (p < 0.01 for paired student t-test; Fig 5). While the variability within each size class is substantial, this result suggests that changes in size class did not drive the observed increases in productivity. Note, while the basal area of a tree scales non-linearly with DBH, BAI is a function of growth in any given year and is independent of tree DBH.

## Discussion

### Plot-level Changes in Biomass with Land Cover Change

We found that aboveground biomass was negatively correlated with development intensity (e.g. OTH < MDR < LDR). Reductions in aboveground biomass with increasing development intensity were likely due to a combination of biomass removal associated with development and ensuing land management practices, including tree maintenance and removal. Hutyra et al. [11] and Raciti et al. [49] found similarly large decreases in aboveground biomass with increasing intensity of urban development. We also found that as development intensity increased, the percentage of paved and lawn land covers increased while the percentage forested cover decreased. Further, plots that underwent a forest-to-OTH land cover change typically had fewer and smaller trees, and consequently far less aboveground biomass than the other urban categories. While we observed a ~100% increase in tree growth rates following development, we found that there was little relationship between plot biomass and growth rates. The biomass present within a plot at any point in time is a product of growth, recruitment, planting, and mortality rates integrated over time. Further, tree growth rates reflect both inherent biological processes and active land management. Since these processes and management choices likely change through time, we were only able to capture the current aboveground biomass pools and the pre- and post- development growth rates for one tree species (red oak).

We found no significant relationship between plot-level ISA and biomass. The ISA-biomass relationship was influenced by plots that simultaneously contained high canopy cover (approaching 100%), high biomass, and high ISA due to a small number of large trees whose expansive crowns overtopped the paved areas around them. This result is in contrast to the significant decreases of biomass with increases in ISA found by Raciti et al. [49] at larger spatial scales.

### Tree-level productivity and land cover change

Tree productivity is a function of many biotic and abiotic factors, including: species, age, resource availability, growing season length, and competition [64,65]. Searle et al. [42] found an eight-fold increase in tree growth rates in red oak seedlings in New York City compared to seedlings in rural New York that they attributed to local temperature differences. Gregg et al. [43] suggested that lower levels of atmospheric ozone in urban areas, compared to adjacent suburban and rural areas, were responsible for elevated urban tree growth rates in New York City *Populus deltoides* seedlings.

Despite a wide range of counterbalancing factors that might lead to increased or decreased vegetation productivity in urban areas, we found that the productivity (in BAI) of individual trees not removed during urbanization doubled with the conversion of forest to urban land cover. While we are not aware of other studies that explicitly quantify tree growth in response to urbanization, several forest gap studies have found that mature red oak trees growing in gaps created in a Connecticut forest during tree harvesting experienced a 25–47% increase in DBH 5–12 years following disturbance [66,67]. Ward [66,67] attributed increased growth rates to crown release, as the trees in those studies had 25–100% of their canopy released, which is likely comparable to the increased light conditions experienced by residual trees on recently developed land. Tree crown release could be responsible for the similar increases in growth observed in our study, as greater than 80% of the trees that we cored were within 10 meters of an edge or clearing, at the edge itself, or were completely isolated from other trees (e.g., in a lawn). Trees growing in these conditions would have been exposed to more light due to less competition and, depending on land management practices, could have also been irrigated, fertilized, and given treatment for pests and diseases.

In order to control for drivers of tree growth other than land cover change, we compared red oak BAI after land cover change with BAI from trees in stable urban conditions (i.e., no known land cover changes since at least 1971), from Boston street trees, and from stable forests (i.e., from FIA plots). Before land cover change, tree growth rates were comparable to FIA forest plots. In contrast, the average growth rates for post-land cover change trees, Boston street trees, and the stable urban trees were statistically indistinguishable from one another, and all were enhanced relative to FIA and pre-disturbance BAI. Elevated light availability is an environmental condition shared by urban trees generally, which may explain the higher growth rates in these locations compared to forest trees.

The suite of observational data from our study is consistent with the hypothesis that reduced competition for light and nutrients after land cover change stimulates tree growth. The timing of tree growth response was closely correlated with our carefully determined date of land cover change, over half of the plots experiencing land cover change showed a large increase in tree growth within five years of the land cover change. In several cases where a strong increase in growth did not fall within the 5-year window of the land cover change date, we suspect that an earlier land cover change may have occurred (see S1 Fig). Further, the increases in growth after land cover change exceeded inter-annual variation. Annual tree ring data can be noisy, but for over 3/4 of all cores analyzed we observed a flat trend (slope ~0) in increment prior to development, followed by a dramatic increase in growth rates after development. In several instances growth rates increased over 1000% after land cover change. We do not know of any other abrupt anthropogenic changes in growing conditions during the 1971–2012 time period, other than the identified land cover change. During this study interval, there were climatological and ecological disturbances, like the 1980 Gypsy Moth infestation. However, the resulting changes in growth associated with these natural disturbances were typically short lived and/or several orders of magnitude smaller than those seen after land cover change.

We observed the strongest growth response in the trees with the slowest growth prior to land cover change. The three trees that did not experience a significant enhancement in growth following development had high pre-development growth rates, suggesting that growing conditions for these trees may have been altered prior to the estimated land cover change date or that resources made available after land cover change were not previously limiting. Further, for two of the three cores that showed no response in growth after development, the estimated land cover change date fell more than three years outside of the identified CCDC/MacConnell land cover change time period (see S1 Fig). This suggests that we may have missed the timing of growth release in the cores that exhibit no change after our estimated date of land cover change.

#### Effect of elevated productivity on carbon stocks in urban areas

While there remains considerable uncertainty regarding C fluxes and terrestrial C stocks in urban areas [68,69], recent studies have suggested that urban vegetation plays an important role in C cycling. Studies that used field and remote sensing data to map vegetation have found extensive canopy cover and large aboveground C stocks in urban areas [70,71]. Furthermore, the vegetative fluxes in urban areas may be increasing due to urbanization-induced changes to regional climate. For example, it has been estimated that gross primary productivity has increased by 13% in the heavily developed eastern U.S., primarily through increases in downwelling shortwave radiation and temperature [10]. Briber et al. [29] estimated that urban heat islands and resultant longer growing seasons may account for as much as a 50% increase in net biogenic exchange in the City of Boston, though there is still large uncertainty regarding the role of growing season length in C sequestration [72].

Despite the substantial extent of urban vegetation and potentially high urban C densities, many remote sensing products and C mapping initiatives discount or completely ignore vegetation in urban areas (e.g. [71,73–75]). It should be noted that many remote sensing products were never intended to map vegetation in urban areas, rather their focus is typically on forested land covers. In the case of the NASA Carbon Management System forest biomass maps for biosphere flux estimation [76], ~ 30% of Massachusetts is effectively assumed to have no vegetative fluxes due to its degree of urbanization. We found that aboveground biomass in urban land covers stored as much as 42.7 ± 14.7 Mg C ha^-1^, which is nearly 1/3 the aboveground C stocks in mature New England forests such as Harvard Forest [77]. These urban C pools coupled with the rapid tree growth observed in this study highlight the importance of urban vegetation for C storage and large biological fluxes from highly developed regions such as the northeastern United States.

In order to explore the role of urban vegetation in net C sequestration, we must further our understanding of urban tree mortality and recruitment/planting rates [78–80]. For example, Nowak et al. [78] found that mortality rates for urban trees were 6.6% yr^-1^, resulting in an overall net decrease in the number of live trees by 4.2% yr^-1^. Urban tree mortality is often higher for smaller, more recently planted trees. Nowak et al. [79] found mortality rates for newly planted street trees can be as high as 19% yr^-1^. However, over 50% of the red oaks that we cored during our 2013 campaign date back to at least 1950, suggesting that mortality may affect street trees more than residual trees left standing or trees planted after land cover change.

Considering only trees large enough for coring in this study (≥ 20 cm in DBH), we found 39.1 ± 7.7 Mg C ha^-1^ of biomass in developed areas and an aboveground biomass growth rate of 1.8 ± 0.4 Mg C ha^-1^ yr^-1^, representing a 4.6% yr^-1^ increase in biomass (ranging from 3.8 to 5.7% yr^-1^). For comparison, we extracted data from the Harvard Forest data archives [81] for a mature mixed-hardwood forest in the Environmental Measurement Site at Harvard Forest in central Massachusetts. Using aboveground biomass accumulation of trees ≥ 20 cm in DBH over a 16 year period (1998 through 2013) we found a 1.4 ± 0.3% yr^-1^ increase in biomass (accounting for recruitment and mortality), with 1.5 ± 0.34 Mg C ha^-1^ yr^-1^ biomass accumulation and 117 ± 7.9 Mg C ha^-1^ in aboveground live biomass [81]. Despite having ~1/3 the aboveground biomass, the biomass growth rate in developed areas is comparable to that of Harvard Forest.

The high growth rates in developed areas likely reflect favorable growing conditions, but this analysis does not fully inform us of the annual vegetative C balance since it is likely that recruitment rates will be lower and mortality rates will be higher within developed areas. Moreover, urban tree biomass estimates in this paper were based on forest allometries which may not accurately reflect urban tree forms [82]. Nonetheless, the high observed tree growth rates highlight that vegetation within developed areas is very dynamic and has the potential to be a significant C sink at some points in time and space.

Our results clearly show that the conversion of forests to developed areas have the potential to considerably alter both ecosystem productivity and the regional C balance. It is likely that urban ecosystems could sequester a significant amount of C, depending on residential and municipal landscape management choices. Given global forecasts for continued population growth, patterns of urban expansion and the resulting land cover changes, urban areas will continue to be a critical component of the changing global carbon cycle [68]. Additional measurements of vegetation growth, mortality, planting, and recruitment rates within developed areas will be necessary for constraining our estimates of urban regional C balance and informing our land development choices. Moreover, more accurate urban tree allometries are needed to better estimate terrestrial C stocks in urban areas. The consequences of development will vary among pre-development ecosystems (e.g., forest vs. grassland) and land use conversion pathways (e.g., forest to development vs. agriculture to development), improved our global estimates of both land cover change and biomass are needed to assess the C impacts of different development typologies [83].

## Supporting Information

S1 Fig Raw increment data. Time series of basal area increment for each individual core in our analysis. The header at the top of each page indicates the type of core. The cores under the “Strong Response” (≥ 200% increase in BAI), “Typical Response” (statically significant increase in BAI), and “Negative/No Response” (statistically significant decrease in BAI or no significant change in BAI) headers comprise the cores from trees where known land cover changed occurred. For these cores, the orange vertical line indicates the estimated disturbance date and the grey area indicates the time period over which the land cover change should have taken place, according to a combination of the CCDC and MacConnell datalayers. Before and after land cover change mean basal area increment is displayed above the plots along with the p-value for the difference between these means (95% confidence). Also included are the cores from the “Stable Urban” and Boston “Street Tree” locations for comparison. Mean basal area increment for the entire time series is displayed above the plots.
(PDF)

S1 Table Plot coordinates. A table of all plots used in this analysis, with Massachusetts town name, longitude, latitude, and the number of samples per plot. Note that FIA data locations were confidential, and were not released. For those plots, data were grouped by county.
(XLSX)
