# Supplementary material for: Tree Productivity Enhanced with Conversion from Forest to Urban Land Covers
Source: PLoS One. 2015 Aug 24;10(8):e0136237. doi: 10.1371/journal.pone.0136237 (PMC4547753; doi:10.1371/journal.pone.0136237)
Supplement: S1 Fig — Time series of basal area increment for each individual core in our analysis. The header at the top of each page indicates the type of core. The cores under the “Strong Response” (≥ 200% increase in BAI), “Typical Response” (statically significant increase in BAI), and “Negative/No Response” (statistically significant decrease in BAI or no significant change in BAI) headers comprise the cores from trees where known land cover changed occurred. For these cores, the orange vertical line indicates the estimated disturbance date and the grey area indicates the time period over which the land cover change should have taken place, according to a combination of the CCDC and MacConnell datalayers. Before and after land cover change mean basal area increment is displayed above the plots along with the p-value for the difference between these means (95% confidence). Also included are the cores from the “Stable Urban” and Boston “Street Tree” locations for comparison. Mean basal area increment for the entire time series is displayed above the plots. (PDF) [file pone.0136237.s001.pdf]

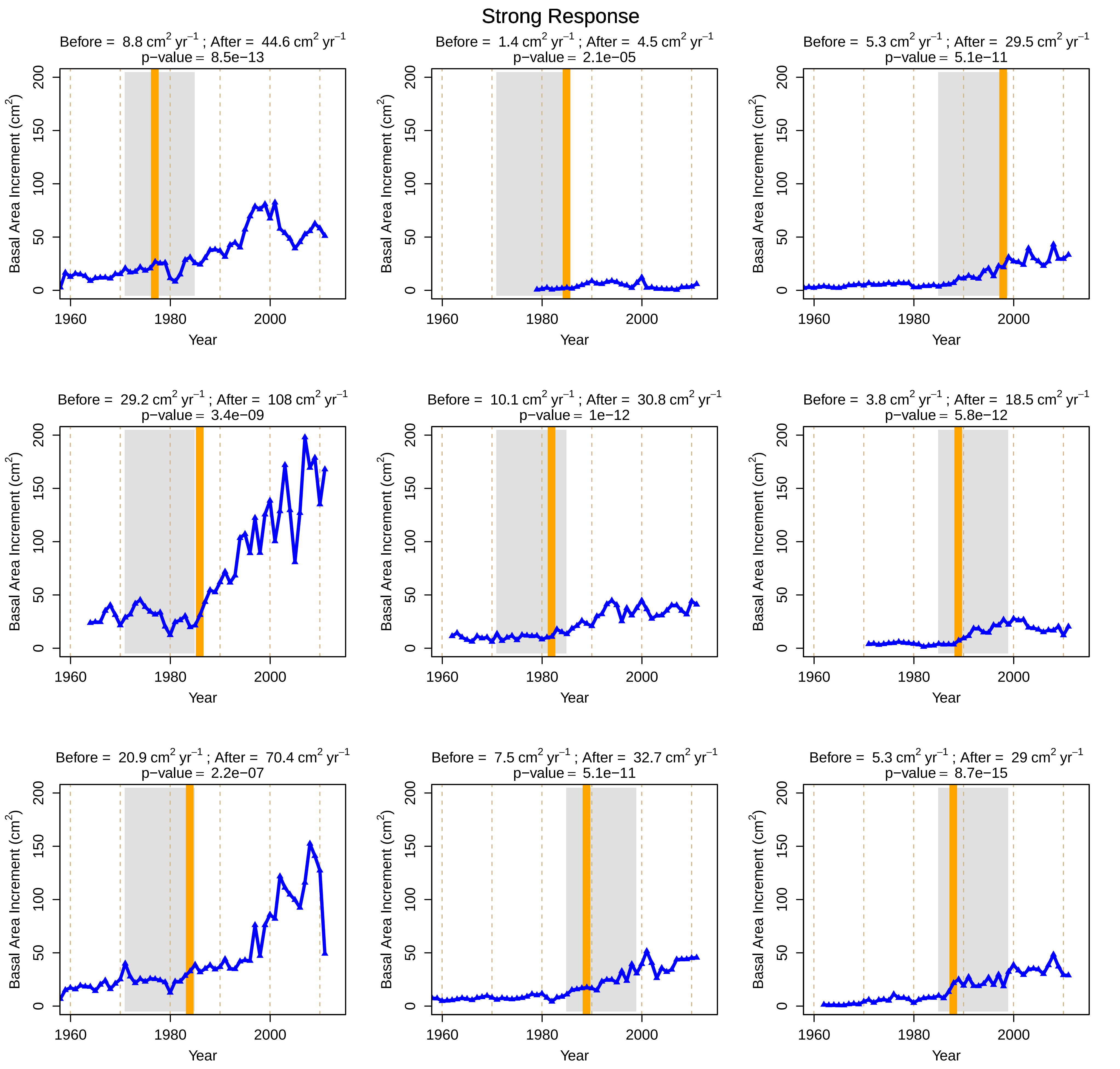
Strong Response
Before = 8.8 cm2 yr−1 ; After = 44.6 cm2 yr−1
p−value = 8.5e−13
Before = 1.4 cm2 yr−1 ; After = 4.5 cm2 yr−1
p−value = 2.1e−05
Before = 5.3 cm2 yr−1 ; After = 29.5 cm2 yr−1
p−value = 5.1e−11
Before = 29.2 cm2 yr−1 ; After = 108 cm2 yr−1
p−value = 3.4e−09
Before = 10.1 cm2 yr−1 ; After = 30.8 cm2 yr−1
p−value = 1e−12
Before = 3.8 cm2 yr−1 ; After = 18.5 cm2 yr−1
p−value = 5.8e−12
Before = 20.9 cm2 yr−1 ; After = 70.4 cm2 yr−1
p−value = 2.2e−07
Before = 7.5 cm2 yr−1 ; After = 32.7 cm2 yr−1
p−value = 5.1e−11
Before = 5.3 cm2 yr−1 ; After = 29 cm2 yr−1
p−value = 8.7e−15
Basal Area Increment (cm2)
0
50
100
150
200
1960
1980
2000
Year

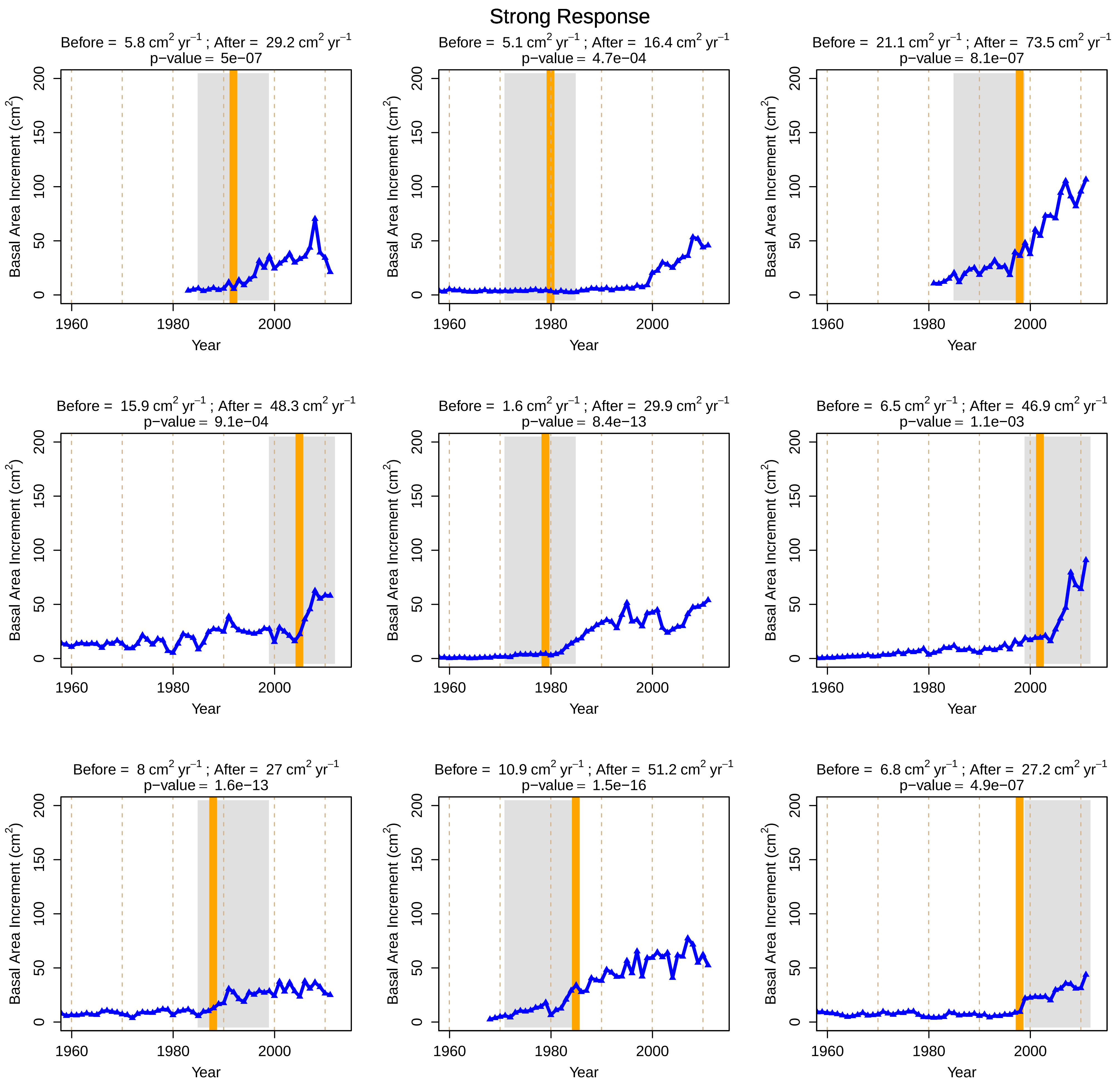
Strong Response
Before = 5.8 cm^2 yr^{-1} ; After = 29.2 cm^2 yr^{-1}
p−value = 5e−07
Before = 5.1 cm^2 yr^{-1} ; After = 16.4 cm^2 yr^{-1}
p−value = 4.7e−04
Before = 21.1 cm^2 yr^{-1} ; After = 73.5 cm^2 yr^{-1}
p−value = 8.1e−07
Before = 15.9 cm^2 yr^{-1} ; After = 48.3 cm^2 yr^{-1}
p−value = 9.1e−04
Before = 1.6 cm^2 yr^{-1} ; After = 29.9 cm^2 yr^{-1}
p−value = 8.4e−13
Before = 6.5 cm^2 yr^{-1} ; After = 46.9 cm^2 yr^{-1}
p−value = 1.1e−03
Before = 8 cm^2 yr^{-1} ; After = 27 cm^2 yr^{-1}
p−value = 1.6e−13
Before = 10.9 cm^2 yr^{-1} ; After = 51.2 cm^2 yr^{-1}
p−value = 1.5e−16
Before = 6.8 cm^2 yr^{-1} ; After = 27.2 cm^2 yr^{-1}
p−value = 4.9e−07
Basal Area Increment (cm^2)
0
50
100
150
200
Year
1960
1980
2000

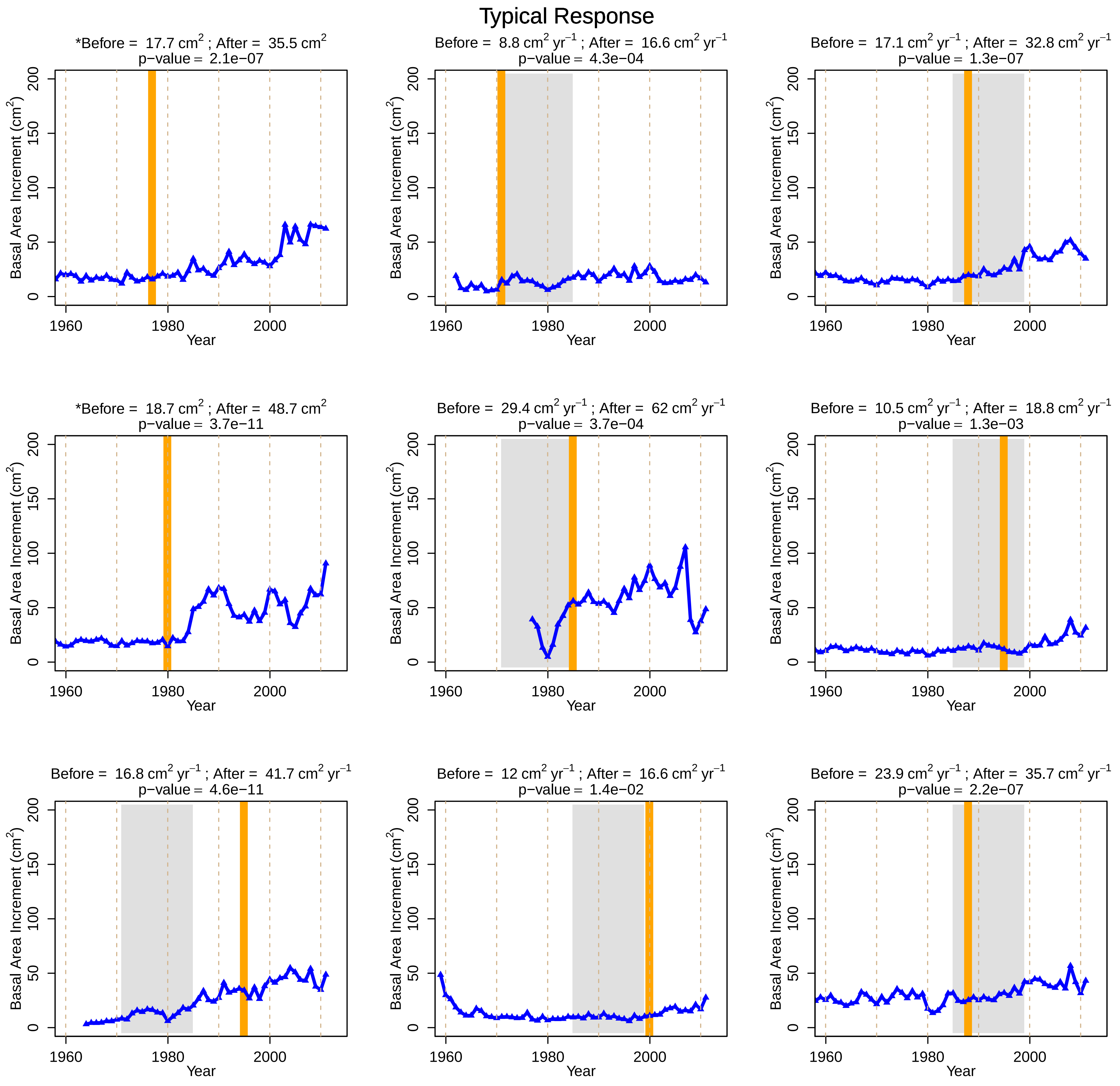

Typical Response
*Before = 17.7 cm2 ; After = 35.5 cm2
p−value = 2.1e−07
Before = 8.8 cm2 yr−1 ; After = 16.6 cm2 yr−1
p−value = 4.3e−04
Before = 17.1 cm2 yr−1 ; After = 32.8 cm2 yr−1
p−value = 1.3e−07
*Before = 18.7 cm2 ; After = 48.7 cm2
p−value = 3.7e−11
Before = 29.4 cm2 yr−1 ; After = 62 cm2 yr−1
p−value = 3.7e−04
Before = 10.5 cm2 yr−1 ; After = 18.8 cm2 yr−1
p−value = 1.3e−03
Before = 16.8 cm2 yr−1 ; After = 41.7 cm2 yr−1
p−value = 4.6e−11
Before = 12 cm2 yr−1 ; After = 16.6 cm2 yr−1
p−value = 1.4e−02
Before = 23.9 cm2 yr−1 ; After = 35.7 cm2 yr−1
p−value = 2.2e−07
Basal Area Increment (cm2)
Year

Typical Response

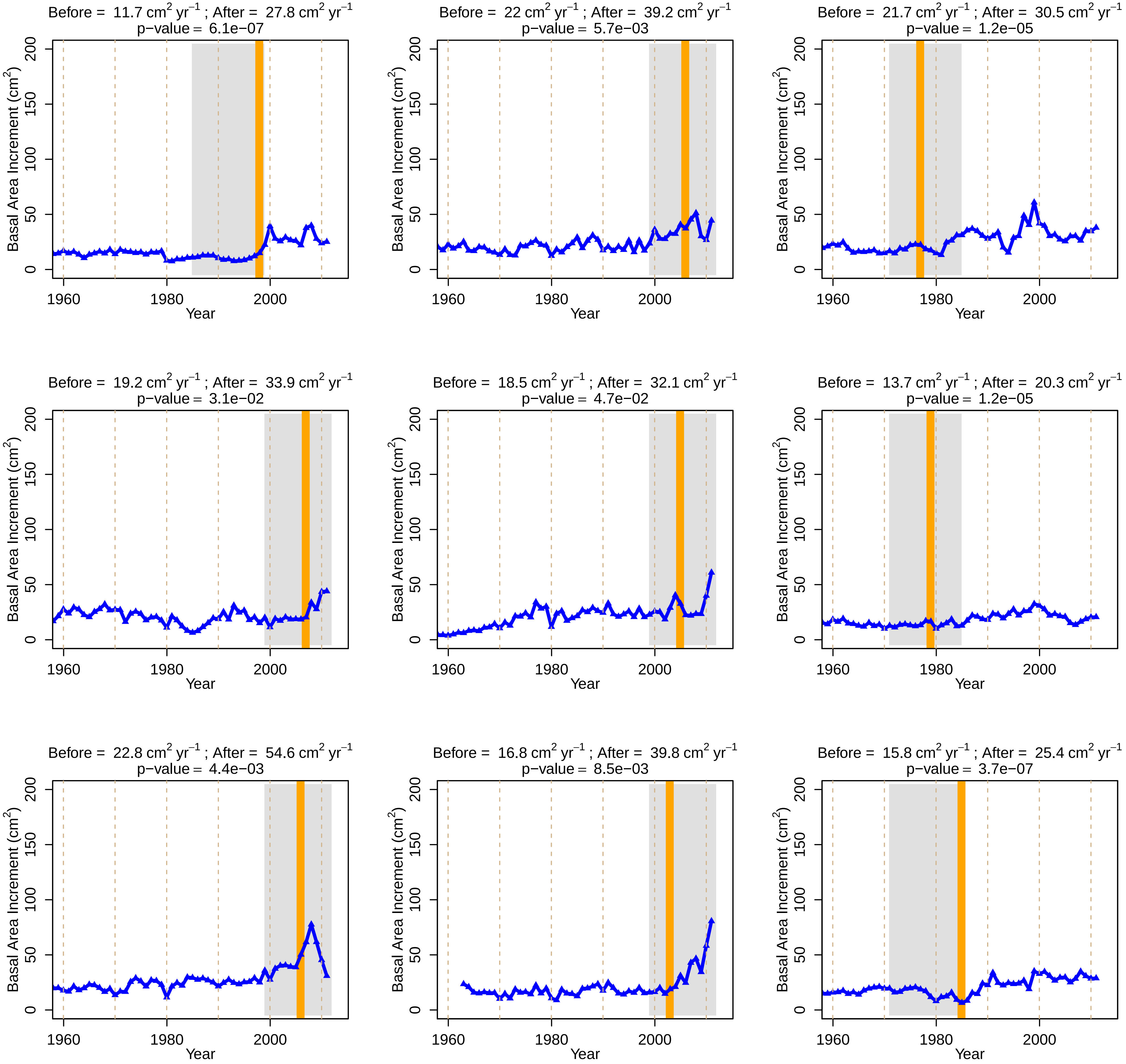

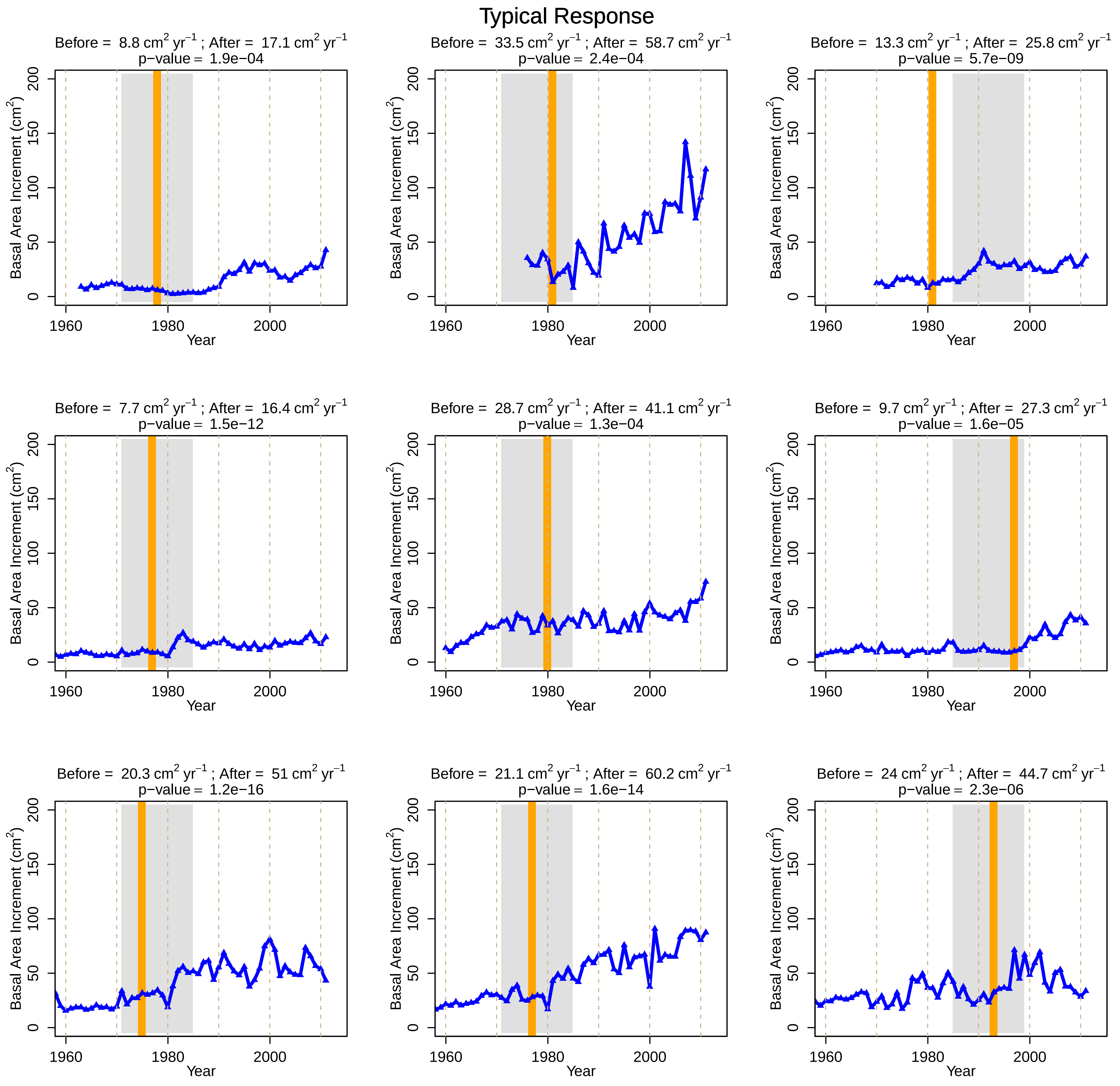
Typical Response
Before = 8.8 cm2 yr−1 ; After = 17.1 cm2 yr−1
p−value = 1.9e−04
Before = 33.5 cm2 yr−1 ; After = 58.7 cm2 yr−1
p−value = 2.4e−04
Before = 13.3 cm2 yr−1 ; After = 25.8 cm2 yr−1
p−value = 5.7e−09
Before = 7.7 cm2 yr−1 ; After = 16.4 cm2 yr−1
p−value = 1.5e−12
Before = 28.7 cm2 yr−1 ; After = 41.1 cm2 yr−1
p−value = 1.3e−04
Before = 9.7 cm2 yr−1 ; After = 27.3 cm2 yr−1
p−value = 1.6e−05
Before = 20.3 cm2 yr−1 ; After = 51 cm2 yr−1
p−value = 1.2e−16
Before = 21.1 cm2 yr−1 ; After = 60.2 cm2 yr−1
p−value = 1.6e−14
Before = 24 cm2 yr−1 ; After = 44.7 cm2 yr−1
p−value = 2.3e−06
Basal Area Increment (cm2)
Year
0
50
100
150
200
1960
1980
2000

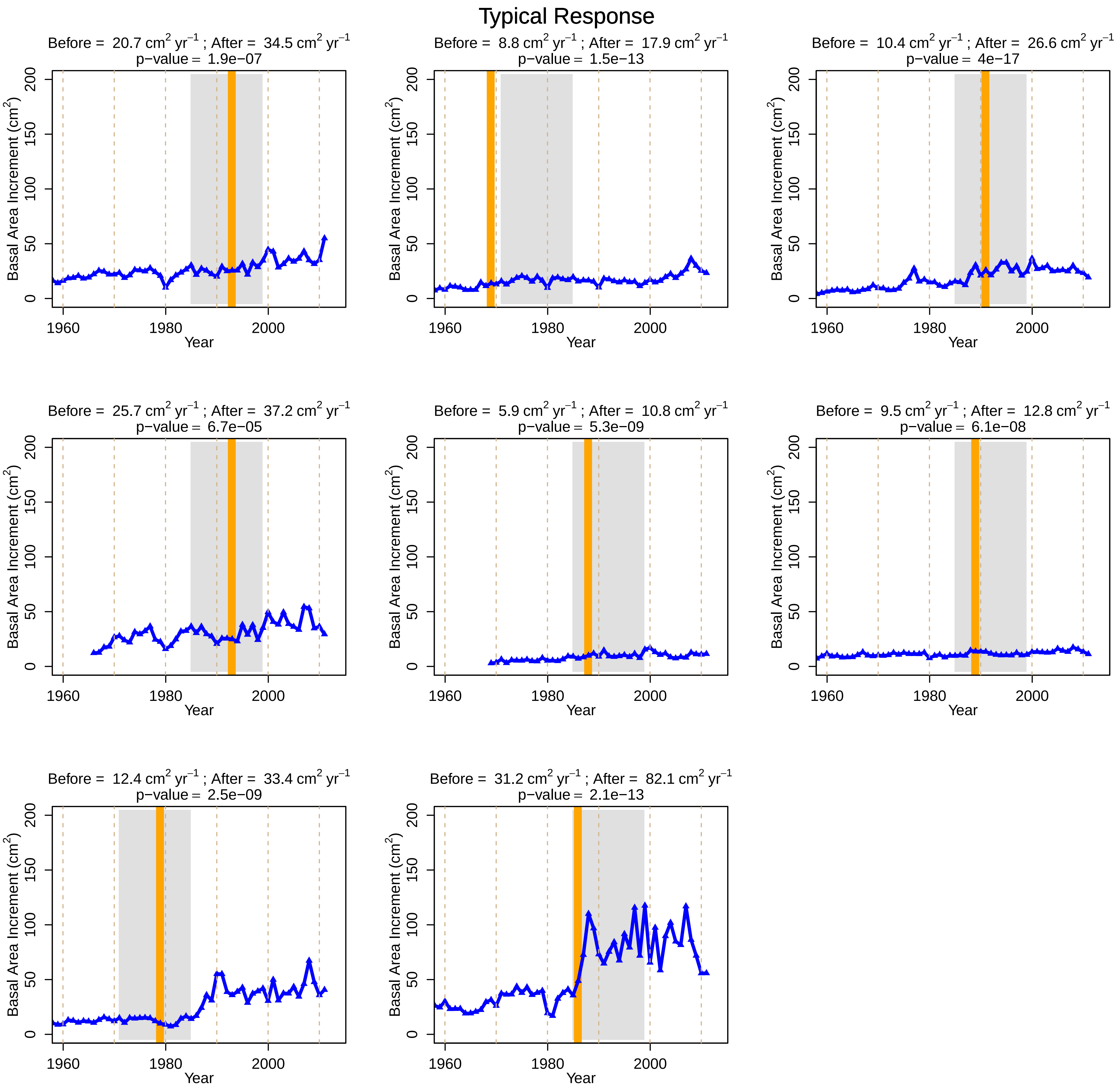

Typical Response
Before = 20.7 cm^2 yr^{-1} ; After = 34.5 cm^2 yr^{-1}
p−value = 1.9e−07
Before = 8.8 cm^2 yr^{-1} ; After = 17.9 cm^2 yr^{-1}
p−value = 1.5e−13
Before = 10.4 cm^2 yr^{-1} ; After = 26.6 cm^2 yr^{-1}
p−value = 4e−17
Before = 25.7 cm^2 yr^{-1} ; After = 37.2 cm^2 yr^{-1}
p−value = 6.7e−05
Before = 5.9 cm^2 yr^{-1} ; After = 10.8 cm^2 yr^{-1}
p−value = 5.3e−09
Before = 9.5 cm^2 yr^{-1} ; After = 12.8 cm^2 yr^{-1}
p−value = 6.1e−08
Before = 12.4 cm^2 yr^{-1} ; After = 33.4 cm^2 yr^{-1}
p−value = 2.5e−09
Before = 31.2 cm^2 yr^{-1} ; After = 82.1 cm^2 yr^{-1}
p−value = 2.1e−13
Basal Area Increment (cm^2)
Year
0
50
100
150
200
1960
1980
2000

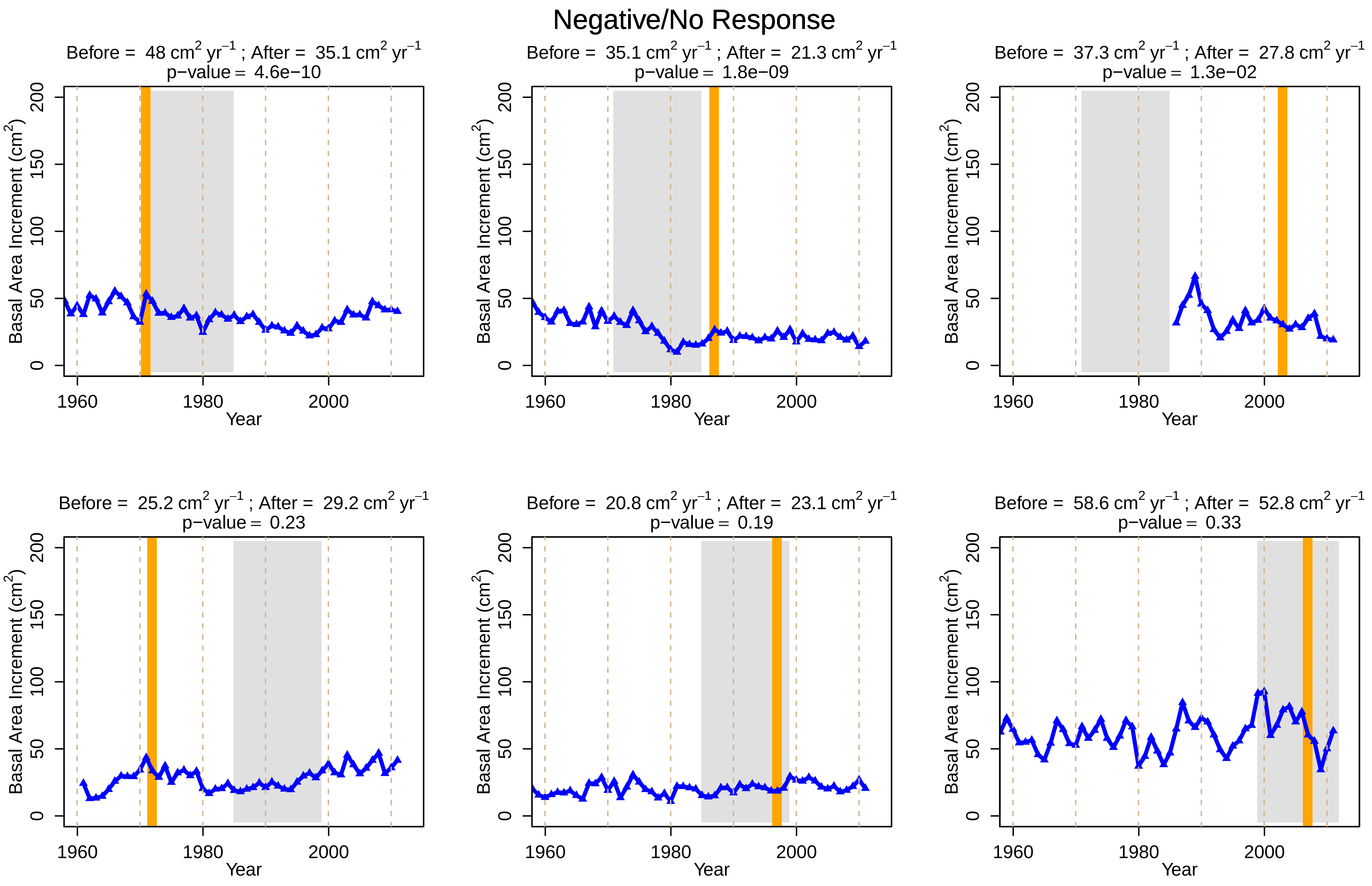

Negative/No Response
Before = 48 cm^2 yr^-1 ; After = 35.1 cm^2 yr^-1
p−value = 4.6e−10
Before = 35.1 cm^2 yr^-1 ; After = 21.3 cm^2 yr^-1
p−value = 1.8e−09
Before = 37.3 cm^2 yr^-1 ; After = 27.8 cm^2 yr^-1
p−value = 1.3e−02
Before = 25.2 cm^2 yr^-1 ; After = 29.2 cm^2 yr^-1
p−value = 0.23
Before = 20.8 cm^2 yr^-1 ; After = 23.1 cm^2 yr^-1
p−value = 0.19
Before = 58.6 cm^2 yr^-1 ; After = 52.8 cm^2 yr^-1
p−value = 0.33
Basal Area Increment (cm^2)
Year
0
50
100
150
200
1960
1980
2000

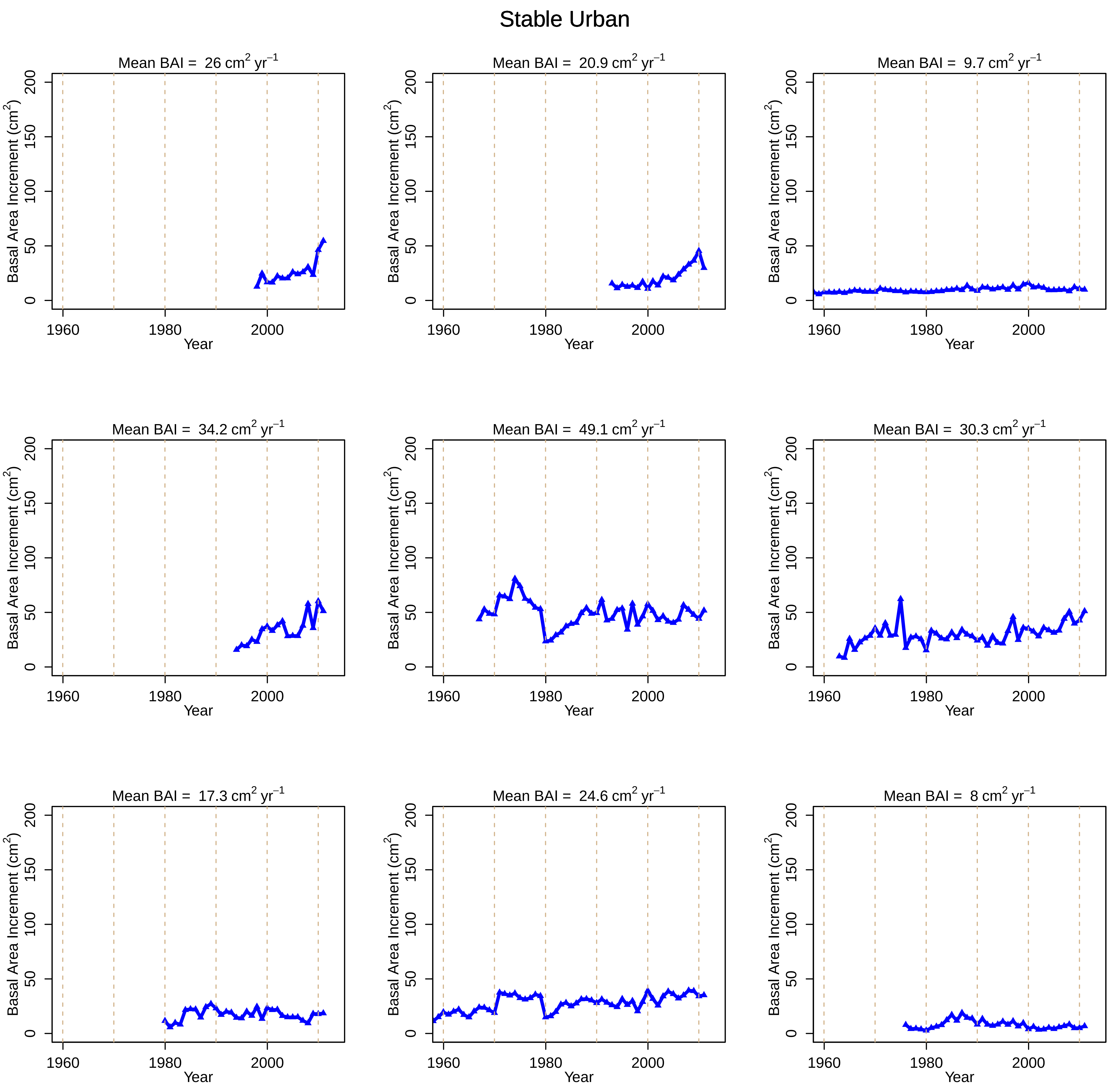
Stable Urban
Mean BAI = 26 cm^2 yr^{-1}
Mean BAI = 20.9 cm^2 yr^{-1}
Mean BAI = 9.7 cm^2 yr^{-1}
Mean BAI = 34.2 cm^2 yr^{-1}
Mean BAI = 49.1 cm^2 yr^{-1}
Mean BAI = 30.3 cm^2 yr^{-1}
Mean BAI = 17.3 cm^2 yr^{-1}
Mean BAI = 24.6 cm^2 yr^{-1}
Mean BAI = 8 cm^2 yr^{-1}
Basal Area Increment (cm^2)
0
50
100
150
200
Year
1960
1980
2000

Stable Urban

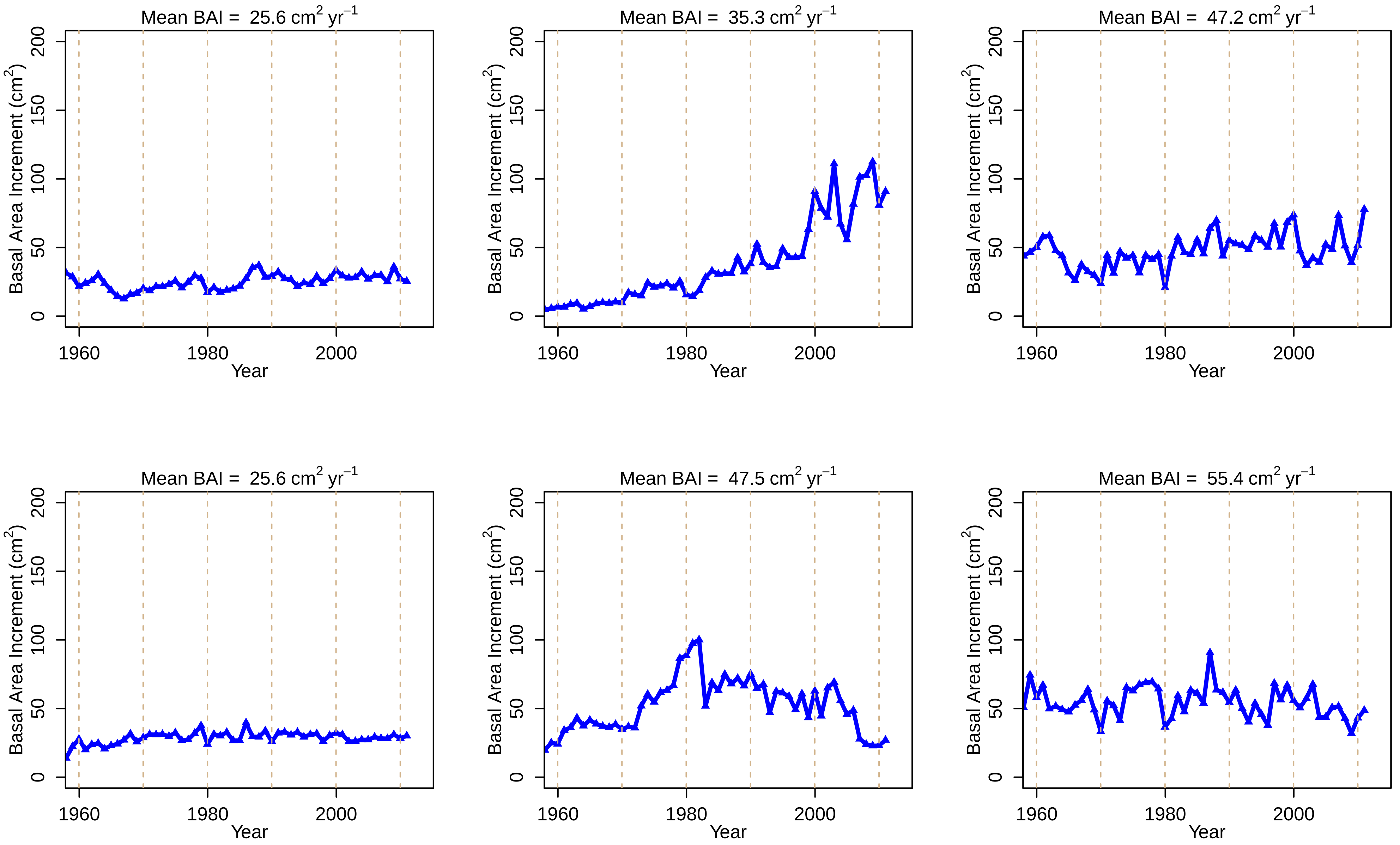

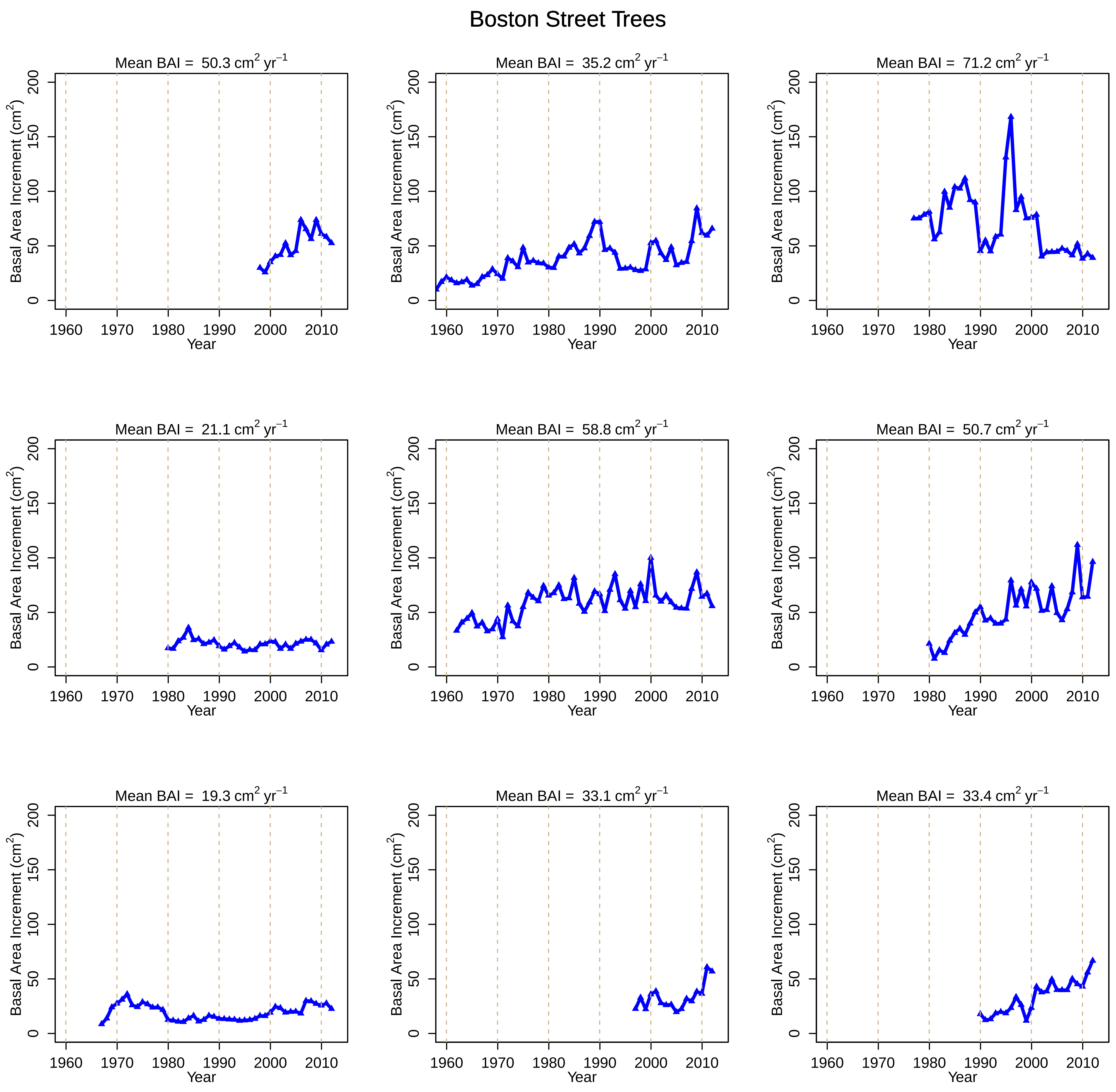
Boston Street Trees
Mean BAI = 50.3 cm^2 yr^{-1}
Mean BAI = 35.2 cm^2 yr^{-1}
Mean BAI = 71.2 cm^2 yr^{-1}
Mean BAI = 21.1 cm^2 yr^{-1}
Mean BAI = 58.8 cm^2 yr^{-1}
Mean BAI = 50.7 cm^2 yr^{-1}
Mean BAI = 19.3 cm^2 yr^{-1}
Mean BAI = 33.1 cm^2 yr^{-1}
Mean BAI = 33.4 cm^2 yr^{-1}
Basal Area Increment (cm^2)
0
50
100
150
200
Year
1960
1970
1980
1990
2000
2010

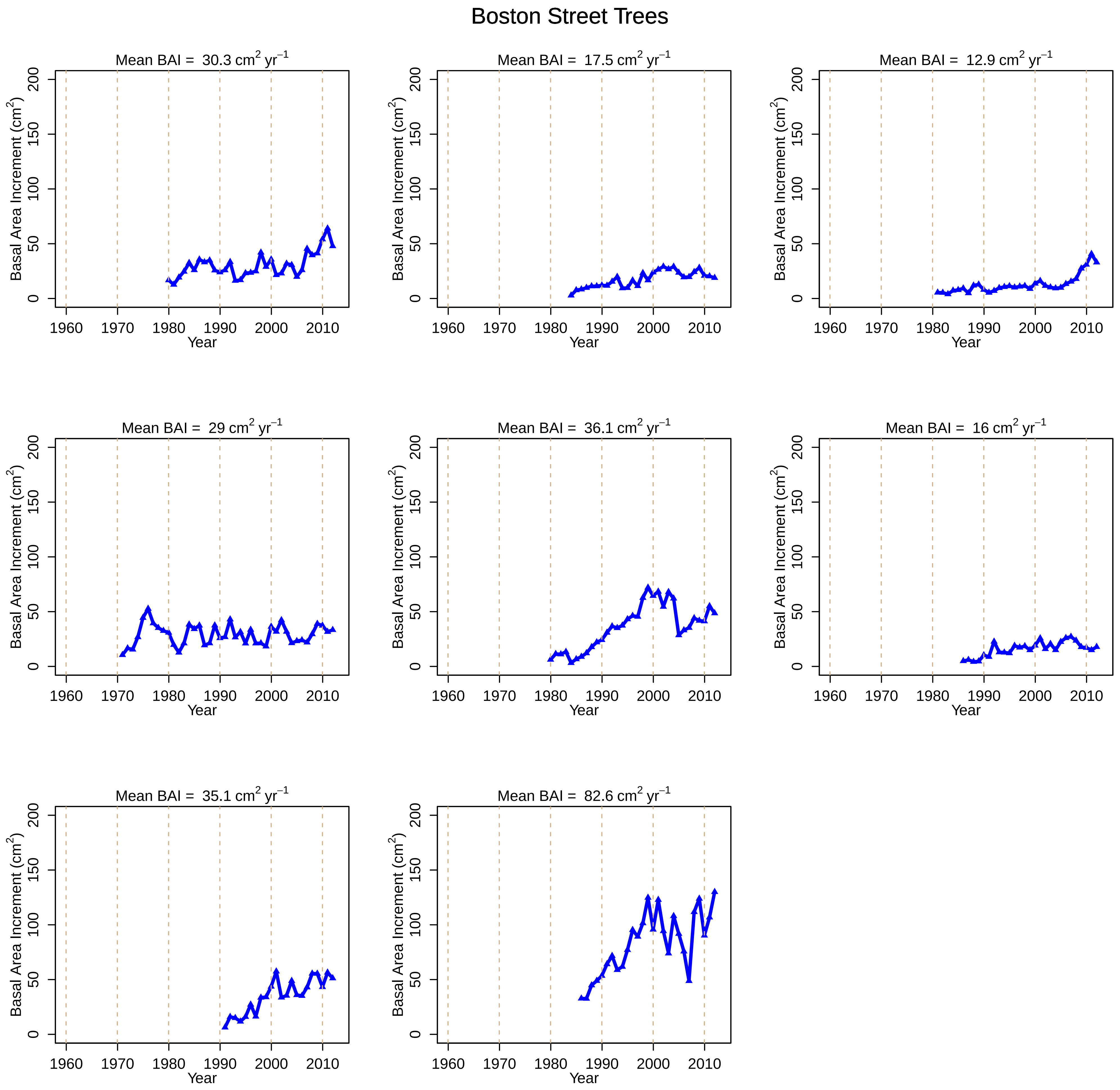
Boston Street Trees
Mean BAI = 30.3 cm2 yr−1
Mean BAI = 17.5 cm2 yr−1
Mean BAI = 12.9 cm2 yr−1
Mean BAI = 29 cm2 yr−1
Mean BAI = 36.1 cm2 yr−1
Mean BAI = 16 cm2 yr−1
Mean BAI = 35.1 cm2 yr−1
Mean BAI = 82.6 cm2 yr−1
Basal Area Increment (cm2)
Year
0
50
100
150
200
1960
1970
1980
1990
2000
2010
